# Primate lentiviruses use at least three alternative strategies to suppress NF-κB-mediated immune activation

**DOI:** 10.1371/journal.ppat.1006598

**Published:** 2017-08-31

**Authors:** Dominik Hotter, Teresa Krabbe, Elisabeth Reith, Ali Gawanbacht, Nadia Rahm, Ahidjo Ayouba, Benoît Van Driessche, Carine Van Lint, Martine Peeters, Frank Kirchhoff, Daniel Sauter

**Affiliations:** 1 Institute of Molecular Virology, Ulm University Medical Center, Ulm, Germany; 2 Institute of Microbiology, Lausanne University Hospital and University of Lausanne, Lausanne, Switzerland; 3 UMI 233 TransVIHMI/INSERM1175, Institut de Recherche pour le Développement (IRD), University of Montpellier, Montpellier, France; 4 Institute for Molecular Biology and Medicine, University of Brussels (ULB), Gosselies, Belgium; Fred Hutchinson Cancer Research Center, UNITED STATES

## Abstract

Primate lentiviruses have evolved sophisticated strategies to suppress the immune response of their host species. For example, HIV-2 and most simian immunodeficiency viruses (SIVs) use their accessory protein Nef to prevent T cell activation and antiviral gene expression by downmodulating the T cell receptor CD3. This Nef function was lost in HIV-1 and other *vpu*-encoding viruses suggesting that the acquisition of Vpu-mediated NF-κB inhibition reduced the selection pressure for inhibition of T cell activation by Nef. To obtain further insights into the modulation of NF-κB activity by primate lentiviral accessory factors, we analyzed 32 Vpr proteins from a large panel of divergent primate lentiviruses. We found that those of SIVcol and SIVolc infecting *Colobinae* monkeys showed the highest efficacy in suppressing NF-κB activation. Vpr-mediated inhibition of NF-κB resulted in decreased IFNβ promoter activity and suppressed type I IFN induction in virally infected primary cells. Interestingly, SIVcol and SIVolc differ from all other primate lentiviruses investigated by the lack of both, a *vpu* gene and efficient Nef-mediated downmodulation of CD3. Thus, primate lentiviruses have evolved at least three alternative strategies to inhibit NF-κB-dependent immune activation. Functional analyses showed that the inhibitory activity of SIVolc and SIVcol Vprs is independent of DCAF1 and the induction of cell cycle arrest. While both Vprs target the IKK complex or a factor further downstream in the NF-κB signaling cascade, only SIVolc Vpr stabilizes IκBα and inhibits p65 phosphorylation. Notably, only *de-novo* synthesized but not virion-associated Vpr suppressed the activation of NF-κB, thus enabling NF-κB-dependent initiation of viral gene transcription during early stages of the replication cycle, while minimizing antiviral gene expression at later stages. Our findings highlight the key role of NF-κB in antiviral immunity and demonstrate that primate lentiviruses follow distinct evolutionary paths to modulate NF-κB-dependent expression of viral and antiviral genes.

## Author summary

The cellular transcription factor NF-κB plays a complex role in the lentiviral replication cycle. On the one hand, activation of NF-κB is required for efficient transcription of viral genes and reactivation of latent proviruses. On the other hand, NF-κB is also a key driver of antiviral gene expression, immune activation and progression to AIDS. As a result, primate lentiviruses tightly regulate the activation of NF-κB throughout their replication cycle to enable transcription of viral genes while minimizing antiviral gene expression. Here, we show that human and simian immunodeficiency viruses have evolved at least three alternative strategies to suppress NF-κB-dependent immune activation: HIV-2 and most SIVs prevent T cell activation via Nef-mediated downmodulation of CD3. In comparison, HIV-1 and its *vpu*-containing SIV precursors inhibit NF-κB activation via their accessory protein Vpu and lost the CD3 downmodulation function of Nef. Finally, SIVcol and SIVolc, infecting mantled guerezas and olive colobus monkeys, respectively, utilize Vpr. Our findings emphasize the key role of NF-κB as inducer of antiretroviral immune responses and add to the accumulating evidence that lentiviral accessory proteins target innate signaling cascades by sophisticated mechanisms to evade restriction.

## Introduction

Cells are equipped with a plethora of pattern recognition receptors (PRRs) that induce an antiviral immune response upon sensing of patterns associated with viral infection. Among them are cytosolic receptors such as cGAS, PQBP1, IFI16, and RIG-I recognizing viral DNA or RNA species, as well as restriction factors such as TRIM5α and Tetherin that sense viral capsids or budding virions, respectively [1]. The signaling cascades initiated by these receptors converge in the activation of a few key transcription factors (*i*.*e*. NF-κB, IRF3 and IRF7), which induce the expression of interferons (IFNs) and other antiviral factors [1,2]. HIV and related simian lentiviruses have evolved sophisticated means to evade these innate sensing pathways. For example, reverse transcription of viral RNA mostly occurs before uncoating of the viral capsid is completed [3]. Thus, viral RNA and DNA intermediates of reverse transcription (RTIs) are cloaked by the viral capsid protein and cellular cofactors to prevent their recognition by cytosolic PRRs [4]. However, primate lentiviruses do not only hide to evade recognition by PRRs, but also directly target the signaling cascades that induce an antiviral immune response. Especially the NF-κB pathway is tightly regulated since this transcription factor plays a dual role in the retroviral replication cycle [5]. While NF-κB is essential for efficient LTR-driven viral gene expression, it may also be detrimental to viral replication because it induces the expression of cellular restriction factors and other antiviral genes. We have recently shown that HIV-1 and related primate lentiviruses cope with this double-edged sword by tight temporal regulation of NF-κB activity throughout the viral replication cycle [6]. During the early stage of infection, the accessory protein Nef boosts NF-κB activation to initiate LTR-driven transcription of viral genes. A similar enhancing effect has been described for the lentiviral envelope glycoprotein gp41 [7]. Once efficient and stable viral transcription is ensured by the viral Tat protein, the late protein Vpu inhibits the activation of NF-κB in a dominant manner to suppress expression of type I IFNs, restriction factors and other antiviral genes [6]. This inhibitory effect on NF-κB is highly conserved among primate lentiviral Vpu proteins [6,8], suggesting an important role *in vivo*. Notably, however, only HIV-1 and a few closely related simian immunodeficiency viruses (SIVs) encode a *vpu* gene. HIV-2 and most SIVs employ an alternative strategy and suppress T cell activation and consequently NF-κB activation and antiviral gene expression by Nef-mediated downmodulation of the T cell receptor (TCR) CD3 from the cell surface [9–11]. This Nef function was lost in HIV-1 and its *vpu*-containing SIV counterparts, *i*.*e*. SIVcpz, SIVgor, SIVgsn, SIVmus and SIVmon, infecting chimpanzees, gorillas and greater spot-nosed, mustached and mona monkeys, respectively [10]. The striking concordance between the presence of a *vpu* gene and loss of a specific Nef function suggests that primate lentiviruses might use Nef-mediated downmodulation of CD3 or Vpu-dependent NF-κB inhibition as alternative strategies to suppress antiviral gene expression during late stages of the viral replication cycle [6,10,11]. One exception, however, has been reported: SIVolc infecting olive colobus monkeys does not encode Vpu, but lost the CD3 downmodulation function of Nef [11]. Here, we examined how SIVolc might modulate NF-κB activity and whether additional exceptions exist. We show that SIVcol from mantled guerezas also lacks both, a *vpu* gene and the ability to efficiently downmodulate CD3 via Nef. Comprehensive analyses of Vpr proteins from a large panel of diverse primate lentiviruses demonstrated that those from SIVolc and SIVcol are most effective in inhibiting the activation of NF-κB, thereby suppressing the induction of IFN expression. Thus, primate lentiviruses evolved at least three alternative strategies (via Nef, Vpu or Vpr) to attenuate NF-κB-dependent antiviral immune activation. Our results illustrate the enormous evolutionary flexibility of lentiviral accessory proteins and corroborate the role of NF-κB as a key regulator of antiretroviral immune responses.

## Results

### Nef proteins of SIVolc and SIVcol do not efficiently downmodulate the T cell receptor CD3

Of more than 20 different SIV investigated, SIVolc from olive colobus monkeys (*Procolobus verus*) is the only known *vpu*-deficient primate lentivirus that fails to downmodulate CD3 via Nef [10,11]. All other *vpu*-deficient SIVs, including SIVwrc from Western red colobus monkeys (*Piliocolobus badius badius*), a close relative of SIVolc [12], use their Nef protein to suppress CD3-mediated T cell activation. To determine whether SIVolc is the only exception, we analyzed the CD3 downmodulation activity of SIVcol from mantled guerezas (*Colobus guereza*), whose Nef protein has not been fully characterized so far. SIVcol Nef caught our attention as it fails to downmodulate CD4, but efficiently decreases CXCR4 surface levels, thereby differing from all other lentiviral Nef proteins investigated [13]. Although SIVcol and SIVolc infect Old World monkey species of the *Colobinae* subfamily, they are only distantly related [14].

**Fig 1:**
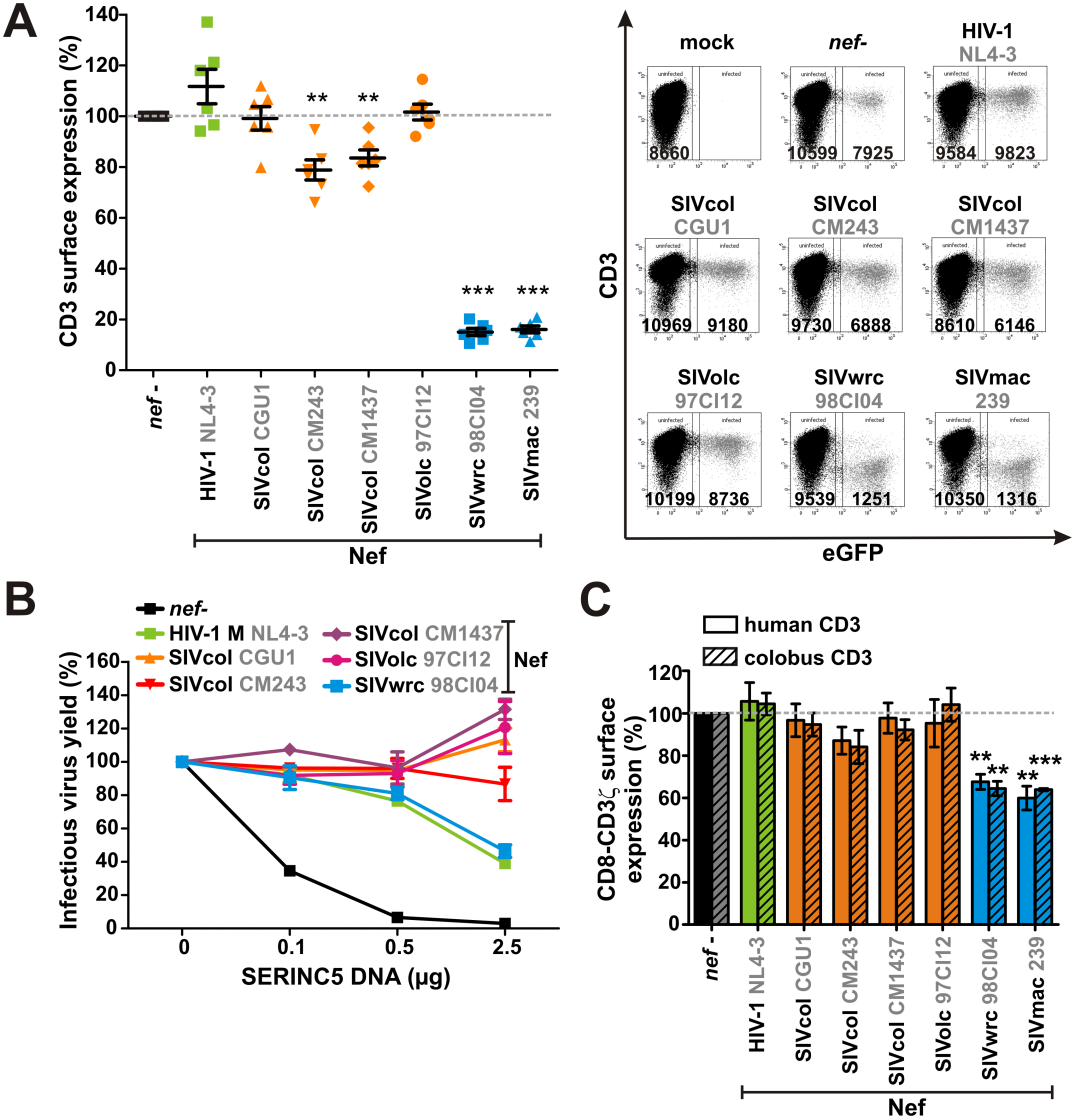
SIVcol and SIVolc Nef fail to efficiently downmodulate the T cell receptor CD3. (A) Human PBMCs were transduced with VSV-G pseudotyped NL4-3 constructs coexpressing the indicated Nef proteins and eGFP via an IRES. 72 hr post-transduction, CD3 surface expression was quantified by flow cytometry. Mean values of six infections ± SEM are shown on the left. The panel on the right shows examples for primary flow cytometry data. (B) Due to the lack of an antibody detecting SIVcol and SIVolc Nef, counteraction of human SERINC5 was analyzed to verify functional Nef expression. HEK293T cells were cotransfected with increasing amounts of a SERINC5 expression plasmid and the NL4-3-based proviral constructs also used in (A). 40 hr post-transfection, infectious virus yield was quantified by infection of TZM-bl reporter cells. Mean values of three independent experiments in triplicates ± SEM are shown. (C) HEK293T cells were cotransfected with the proviral constructs described in (A) and expression vectors for CD3-CD8 fusion proteins comprising the cytoplasmic domain of human or colobus CD3ζ and the extracellular and transmembrane domain of human CD8. 40 hr post transfection, CD3ζ downmodulation was determined by monitoring CD8 surface levels via flow cytometry. Mean values of three to four independent experiments ± SEM are shown. In (A) and (C), asterisks indicate statistically significant differences compared to the *nef*-defective control (**p < 0.01; ***p < 0.001).

CD3 downmodulation was analyzed in human peripheral blood mononuclear cells (PBMCs) infected with HIV-1 NL4-3 IRES eGFP constructs encoding the *nef* alleles of SIVcol isolates CGU1, CM243, and CM1437, SIVolc 97CI12, or SIVwrc 98CI04 (S1A Fig). HIV-1 NL4-3 and SIVmac239 Nefs served as controls. Flow cytometric analyses confirmed [11] that Nef from SIVolc does not downmodulate CD3 (Fig 1A). Interestingly, a similar phenotype was observed for the three SIVcol Nefs, which did not or only inefficiently decrease CD3 cell surface levels in human cells. Notably, all Nef proteins were expressed and functional as they efficiently increased virion infectivity by antagonizing the host restriction factor SERINC5 (Fig 1B) and decreased surface levels of CD28 and/or CXCR4 (S1B and S1C Fig). Since lentiviral Nef proteins target the ζ chain of the TCR complex [15], we PCR-amplified and cloned the CD3ζ chain of colobus monkeys to exclude that lack of activity in SIVcol and SIVolc Nefs is due to species-specific differences in the target sequence. The intracellular domain of human or colobus CD3ζ was fused to the extracellular domain of human CD8 to facilitate detection by flow cytometry. While SIVwrc and SIVmac Nefs downmodulated the human and colobus orthologs with similar efficiencies, SIVolc and SIVcol Nefs had no significant effects (Fig 1C, S1D Fig). Thus, SIVolc and SIVcol lack efficient Nef-mediated suppression of T cell activation, as well as Vpu-mediated inhibition of NF-κB activation, and it remained unclear whether/how they might suppress NF-κB-dependent antiviral gene expression.

### SIVolc and SIVcol Vpr suppress NF-κB activation

**Fig 2:**
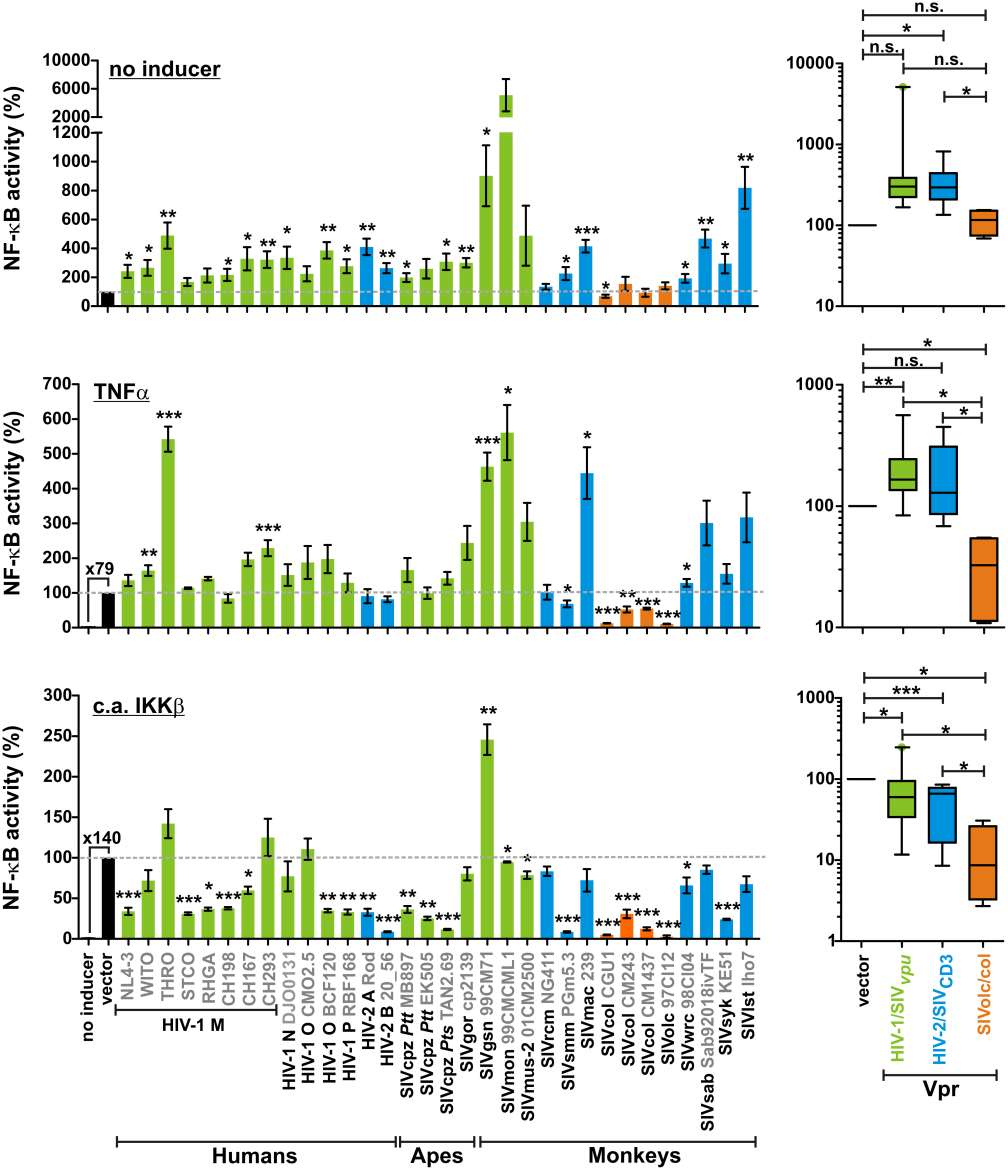
Modulation of NF-κB activity by primate lentiviral Vpr proteins. HEK293T cells were cotransfected with the indicated *vpr* alleles, a firefly luciferase reporter construct under the control of three NF-κB binding sites, and a *Gaussia* luciferase construct for normalization. In the middle and bottom panels, cells were additionally stimulated with TNFα or cotransfected with a constitutively active mutant of IKKβ (c.a. IKKβ), respectively. Luciferase activities were determined 40 hr post-transfection. All values (including the no inducer controls) are shown as percentage of the vector control, which was set to 100%. Mean values of three to ten independent experiments in triplicates ± SEM are shown. Asterisks indicate statistically significant differences compared to the vector control (*p<0.05; **p < 0.01; ***p < 0.001). In the panels on the right, Vprs were divided into three groups: Vprs from lentiviruses encoding *vpu* (HIV/SIV_*vpu*_, green), downmodulating CD3 via Nef (HIV/SIV_CD3_, blue), or lacking a *vpu* gene and the CD3-downmodulation activity (SIVolc/col, orange). Whiskers of the boxplots indicate the 5^th^ and 95^th^ percentiles.

Besides Vpu and Nef, the accessory protein Vpr of the lab-adapted HIV-1 strains 89.6, NL4-3, HxBru, and IIIB_LAI has been suggested to modulate the induction of NF-κB-dependent antiviral and proinflammatory gene expression [16–20]. To obtain more comprehensive insights into the effect of Vpr on NF-κB signaling and immune activation, we examined a panel of 32 phylogenetically diverse *vpr* alleles representing all major lineages of HIV and SIV (*i*.*e*. HIV-1/SIVcpz/SIVgor, HIV-2/SIVsmm/SIVmac, SIVgsn/mon/mus, SIVrcm, SIVsyk, SIVcol, SIVwrc/SIVolc, SIVsab, and SIVlst) [12]. The deduced Vpr amino acid sequences varied considerably in their lengths (83 to 138 amino acids) and showed limited sequence conservation (S2A Fig). One exception was a highly conserved EAxxHF motif in the N-terminal α-helical domain, known to be important for the induction of a G2 cell cycle arrest, nuclear localization and virion-packaging of Vpr [21,22]. To determine the effect of these Vpr proteins on NF-κB activity, we took advantage of a reporter vector expressing firefly luciferase under the control of three NF-κB binding sites [6]. HEK293T cells were cotransfected with this reporter construct, expression plasmids for Vpr, and an NF-κB-independent *Gaussia* luciferase vector to normalize for cell viability and transfection efficiencies. In the absence of any stimulus, most Vpr proteins increased NF-κB activity 2- to 50-fold, with those of SIVgsn, SIVmon and SIVlst having the strongest effects (Fig 2, upper panel). In contrast, Vprs of SIVrcm, SIVcol and SIVolc had no significant effect or even decreased NF-κB activity. While SIVrcm Vpr was hardly detectable by Western blotting, inefficient expression or induction of cell death did not explain the lack of NF-κB modulation by SIVcol and SIVolc Vprs (S2B and S2C Fig).

To further analyze how primate lentiviral Vpr proteins modulate NF-κB activation, we next tested the effects of all Vprs in the presence of TNFα, a potent activator of NF-κB. Again, the effects varied substantially between different Vpr proteins: those from HIV-1 THRO, SIVgsn, SIVmon, SIVmus, SIVmac, SIVsab, and SIVlst enhanced NF-κB-driven reporter gene expression up to 5-fold, whereas SIVolc and SIVcol Vprs suppressed NF-κB activation by about 9-fold (Fig 2, middle panel). To investigate whether the effects of Vpr on NF-κB-mediated gene expression are independent of the receptor, we repeated the experiment using a constitutively active mutant of IKKβ for stimulation. This mutant induces the phosphorylation and degradation of IκBα, the inhibitor of NF-κB, and allows clarifying whether active Vpr proteins target the NF-κB pathway upstream or downstream of the IKK complex. While the impact of lentiviral Vprs on NF-κB activity was again highly variable (Fig 2, bottom panel), the effects correlated well with those observed upon stimulation with TNFα (S2D Fig). However, when IKKβ instead of TNFα was used for stimulation, only SIVgsn Vpr significantly boosted NF-κB activity. Most Vprs displayed modest suppressive effects, while those of SIVcol and SIVolc were highly potent inhibitors (69–95% and 97% reduction, respectively). In line with the high evolutionary conservation of the NF-κB signaling cascade [23], the potent inhibitory effects of SIVcol and SIVolc Vpr were also observed in a simian cell line (S2E and S2F Fig).

To assess whether Vpu or Nef function affected the selection pressure for Vpr-mediated modulation of NF-κB activity, we grouped the Vpr alleles according to the presence or absence of a *vpu* gene and efficient Nef-mediated CD3 downmodulation activity in the respective viruses (Fig 2, right panels). On average, Vpr proteins of lentiviruses expressing Vpu (HIV/SIV_*vpu*_) or downmodulating CD3 (HIV/SIV_CD3_) inhibited NF-κB less efficiently than those of SIVolc/col, or even boosted the activation of this transcription factor. While inhibition of NF-κB may confer a selection advantage as it suppresses antiviral gene expression, primate lentiviruses also need to ensure efficient initiation of NF-κB-dependent viral gene expression. To achieve this, most HIV/SIV strains use Nef and potentially gp41 to boost NF-κB activation during early stages of the replication cycle [6,7]. Some viruses (e.g. SIVgsn, SIVmon), however, lack the ability to enhance NF-κB activation via Nef and may use Vpr instead [6]. Thus, several viral proteins seem to cooperate or compensate for each other in order to regulate the activation of NF-κB throughout the viral replication cycle. Another example is SIVsmm, which induces only low levels of immune activation and apoptosis despite high levels of viremia in infected sooty mangabeys [24]. While this virus prevents T cell activation via Nef-mediated downmodulation of CD3, its Vpr protein also inhibited TNFα- and IKKβ-induced NF-κB activation, albeit to a lesser extent than SIVolc and SIVcol Vpr.

### SIVolc and SIVcol Vpr prevent IFNβ promoter activation by inhibiting NF-κB

**Fig 3:**
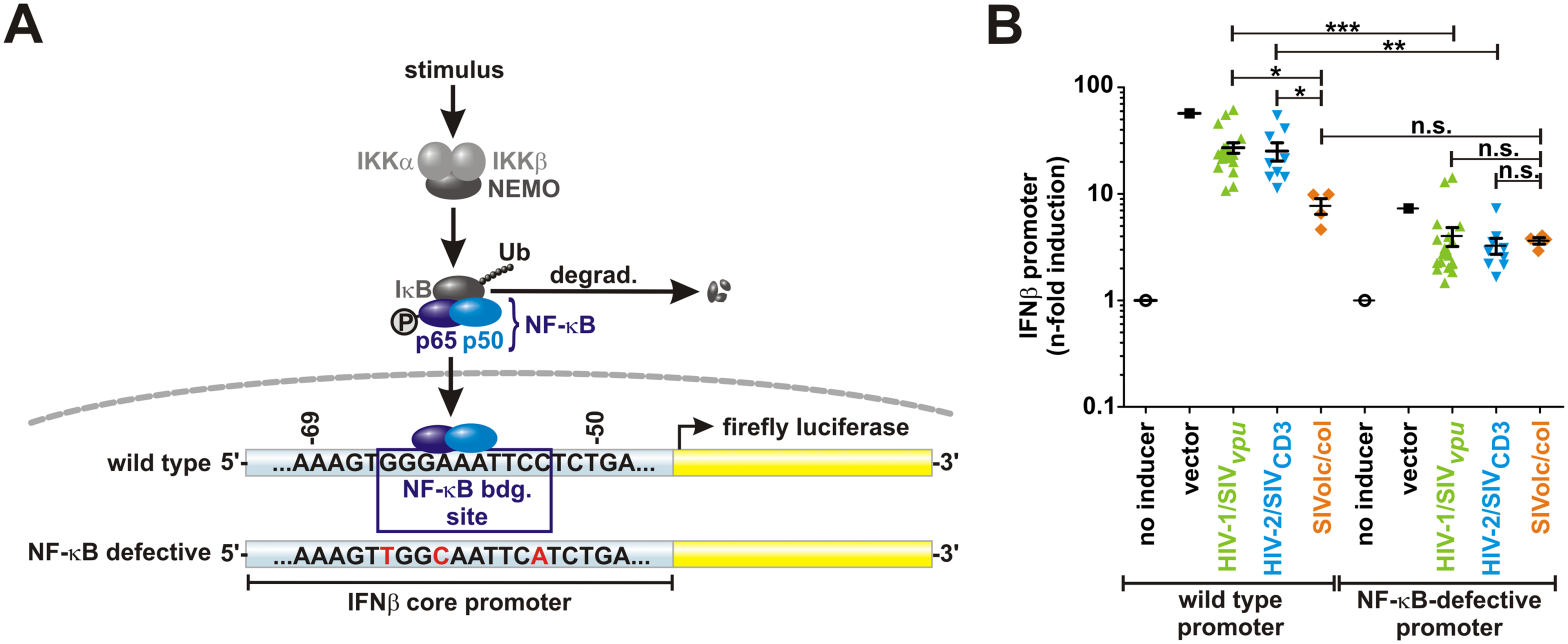
Inhibition of IFNβ promoter activity by SIVcol and SIVolc Vpr. (A) Schematic representation of the canonical NF-κB signaling pathway and the IFNβ promoter reporter constructs used in the present study. The wild type IFNβ core promoter contains an NF-κB binding site that was mutated by introducing three nucleotide changes (highlighted in red). (B) HEK293T cells were cotransfected with different *vpr* alleles, a *Gaussia* luciferase construct for normalization, and one of the firefly luciferase reporter constructs described in (A) (with wild type or mutated NF-κB binding site). To activate the IFNβ promoter, cells were stimulated with Sendai virus. Luciferase activities were determined 40 hr post-transfection. Vprs were categorized into three groups: Vprs from lentiviruses encoding *vpu* (HIV/SIV_*vpu*_, green), downmodulating CD3 via Nef (HIV/SIV_CD3_, blue), or lacking a *vpu* gene and the CD3-downmodulation activity (SIVolc/col, orange) (**p < 0.01; ***p < 0.001; n.s. p>0.05).

To test whether Vpr-mediated modulation of NF-κB activity affects antiviral gene expression, HEK293T cells were cotransfected with expression vectors for Vpr and a reporter construct expressing firefly luciferase under the control of the IFNβ core promoter (Fig 3A). Cells were stimulated with Sendai virus to activate both, NF-κB- and IRF3-mediated transcription [6,25]. Both pathways are evolutionarily highly conserved [23,26,27]. With the exception of a few orthologs (e.g. those of SIVmon, SIVmus, SIVsab), most Vprs suppressed the activation of the IFNβ promoter (S3 Fig). With a >10-fold reduction, SIVolc and SIVcol Vpr were the most potent inhibitors. To determine whether this effect can be attributed to reduced NF-κB activity, the experiment was repeated using a mutant IFNβ promoter lacking the NF-κB binding site (Fig 3A) [6]. Even in the absence of any NF-κB binding site, many Vprs moderately inhibited luciferase reporter gene expression (S3 Fig), suggesting that NF-κB-independent and/or unspecific inhibitory effects contribute to Vpr-mediated suppression of IFNβ induction. On average, however, SIVolc and SIVcol Vprs suppressed IFNβ promoter activity substantially more efficiently in the presence (7.3-fold) than in the absence (2.0-fold) of NF-κB binding sites (Fig 3B). Thus, SIVcol and SIVolc Vpr proteins inhibit virus-induced IFN expression mainly by interfering with NF-κB activation.

### SIVolc Vpr suppresses immune activation in infected T cells

**Fig 4:**
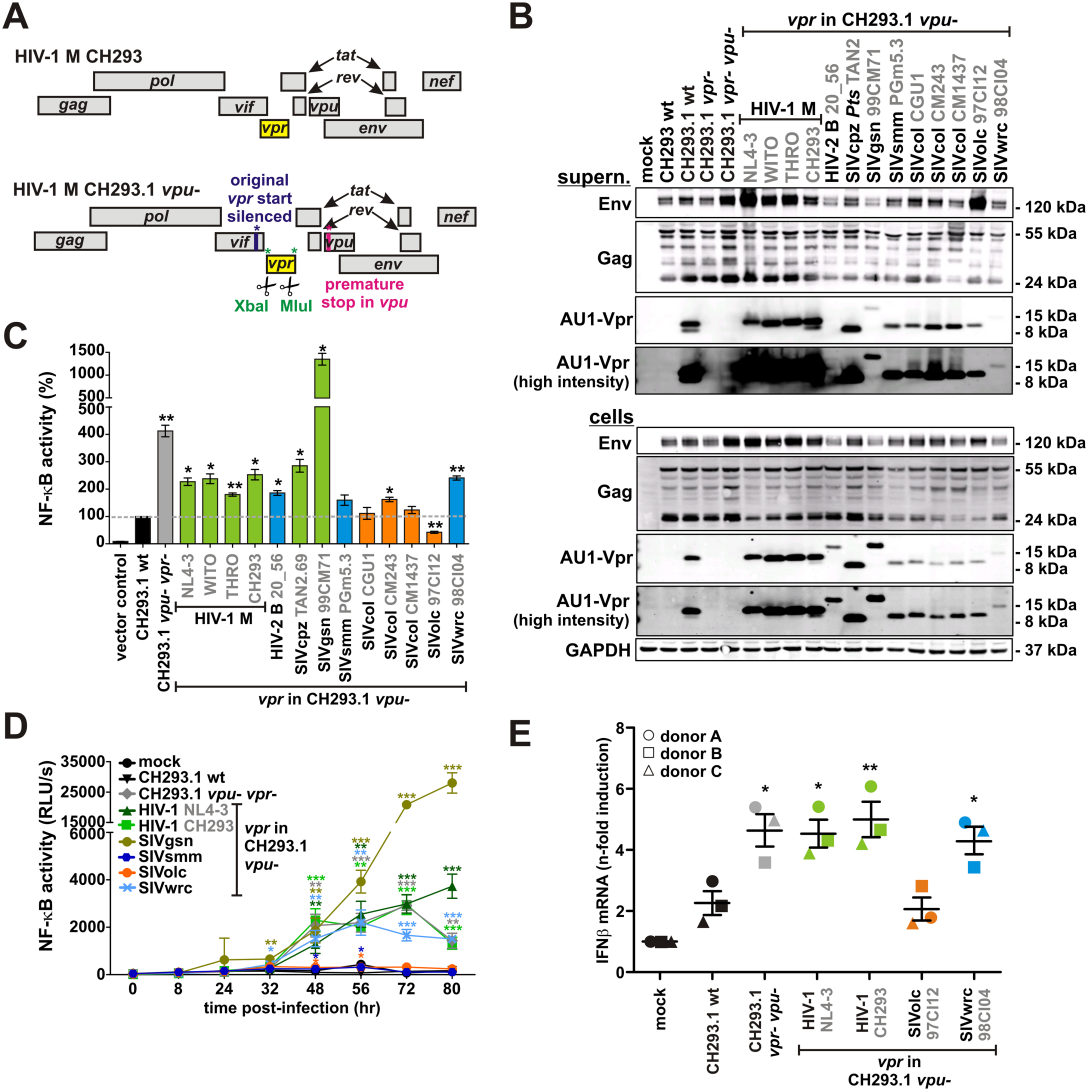
Vpr-mediated modulation of NF-κB activity and immune activation in infected T cells. (A) Genomic organization of a chimeric infectious molecular clone (IMC) of HIV-1 M CH293.1 expressing heterologous *vpr* alleles. The CH293 *vpr* open reading frame was replaced by XbaI and MluI restriction sites (green), allowing the insertion of heterologous AU1-tagged *vpr* alleles (yellow). The original *vpr* start codon in *vif* was silenced (blue) to prevent expression of a truncated CH293 Vpr. Expression of Vpu was abrogated by inserting a premature stop codon (pink). (B) Expression of heterologous Vpr proteins from the CH293.1 chimeras described in (A). HEK293T cells were cotransfected with CH293.1 IMCs encoding the indicated *vpr* alleles. 40 hr post-transfection, cells and supernatants were harvested and virions in the supernatant were purified by centrifugation through a sucrose cushion. Subsequently, Western blotting was performed to detect AU1-tagged Vpr, Env and Gag. Detection of GAPDH served as loading control. (C) HEK293T cells were cotransfected with the indicated CH293.1 chimeras, a firefly luciferase reporter construct under the control of three NF-κB binding sites, and a *Gaussia* luciferase construct for normalization. Luciferase activities were determined 40 hr post-transfection. Mean values of three independent experiments in triplicates ± SEM are shown. (D) The SupD1 NF-κB reporter cell line was transduced with the indicated VSV-G pseudotyped CH293.1 chimeras. Cells were harvested at various time points post-transduction to determine the activation levels of NF-κB. The mean values of triplicate infections ± SD of a representative experiment are shown. (E) PBMCs of three different donors were transduced with VSV-G pseudotyped CH293.1 chimeras encoding the indicated *vpr* alleles. Cells were harvested 72 hr post-transduction and total cellular RNA was isolated and reversely transcribed. IFNβ mRNA levels were determined by quantitative RT-PCR and normalized to GAPDH mRNA. The mean values ± SEM are shown. In (C) to (E), asterisks indicate statistically significant differences compared to CH293.1 wild type transfected or infected cells (*p < 0.05; **p < 0.01; ***p < 0.001).

To analyze modulation of immune activation and IFNβ expression by Vpr in infected cells, we inserted 13 *vpr* alleles with varying effects on NF-κB into the CH293 HIV-1 infectious molecular clone (IMC). We selected CH293 since it represents a primary strain of the most widespread clade C of HIV-1 [28]. The *vpr* open reading frame in the *vif-tat1* intergenic region of HIV-1 M CH293 was replaced by restriction sites for XbaI and MluI, and heterologous AU1-tagged *vpr* alleles were inserted to generate a derivative named CH293.1 (Fig 4A). Premature stop codons were inserted in the *vpu* gene to investigate whether some Vpr proteins may compensate for the lack of Vpu-mediated NF-κB inhibition. In contrast, the *nef* and *env* open reading frames were maintained as the respective gene products have been shown to boost rather than suppress NF-κB activation [6,7]. All proviral constructs expressed their respective heterologous Vpr protein (Fig 4B, lower panel) and (with the exception of HIV-2 20_56) all Vprs were detected in purified viral particles (Fig 4B, upper panel). In some cases (e.g. HIV-2 20_56), exchange of *vpr* reduced Env expression and thus infectious virus yield (Fig 4B, S4A Fig). This defect, however, could be rescued by pseudotyping the virions with the vesicular stomatitis virus glycoprotein (VSV-G) (S4A Fig). Unfortunately, however, the IMCs encoding SIVcol CGU1, CM243 and CM1437 *vpr* remained non-infectious, possibly due to the disruption of splice sites regulating Tat expression [29], and could therefore not be used for infection studies.

All chimeric CH293 constructs were tested in transfected HEK293T cells to elucidate the impact of Vpr on NF-κB activity if expressed in a proviral context, via the viral LTR promoter. Here, the CH293.1 wild type virus induced the activation of NF-κB in the absence of any other stimuli (Fig 4C). This activation was increased about 4-fold in the absence of intact *vpu* and *vpr* genes. Lack of Vpu-mediated NF-κB inhibition was rescued by *in cis* expression of SIVsmm, SIVcol and SIVolc Vpr (Fig 4C). In agreement with the results obtained using expression vectors for Vpr (Fig 2), SIVgsn Vpr boosted the activation of NF-κB. To validate these effects in infected T cells, we transduced SupD1 cells with VSV-G pseudotyped CH293.1 *vpu*- strains expressing the Vpr proteins of HIV-1 NL4-3, SIVgsn, SIVsmm, SIVolc or SIVwrc. This T cell line is a derivative of SupT1 cells and expresses a short-lived firefly luciferase under the control of an NF-κB promoter that allows monitoring of NF-κB activation over time [6]. In agreement with published data [6], *vpu*-defective HIV-1 CH293.1 constructs induced higher levels of NF-κB activation than the parental virus (Fig 4D, S4B Fig). Lack of Vpu-mediated NF-κB inhibition was rescued by SIVsmm and SIVolc (but not by HIV-1 and SIVwrc) Vprs. Again, SIVgsn Vpr enhanced the activation of NF-κB. Notably, these differences in NF-κB activity were not due to differences in infection rates (S4C Fig).

To test whether Vpr-mediated inhibition of NF-κB activation results in attenuated immune activation in primary cells, we infected human PBMCs from three different donors with CH293.1 wt or *vpu*- viruses expressing either no Vpr or the Vpr protein of HIV-1 NL4-3, HIV-1 CH293, SIVolc or SIVwrc. At 72 hours post-infection, IFNβ expression levels were quantified by qRT-PCR. In agreement with potent Vpu-mediated inhibition of NF-κB activation, CH293.1 wild type induced only low levels of IFNβ and deletion of *vpu* and *vpr* led to a 2- to 3-fold increase in IFNβ expression (Fig 4E). This immune activation was fully suppressed by *in cis* complementation with Vpr of SIVolc, but not SIVwrc or HIV-1 Vprs (Fig 4E, S4D Fig). Furthermore, SIVolc Vpr also inhibited induction of IFI44 expression, a typical ISG with proposed anti-HIV-1 activity [30], which was upregulated in PBMCs infected with *vpu*-deficient CH293.1 (S4E Fig). Higher IFNβ and IFI44 levels were not due to increased infection rates (S4F Fig). Thus, SIVolc Vpr is a potent inhibitor of NF-κB-driven immune activation in infected primary cells that can compensate for the lack of *vpu*.

### SIVolc Vpr inhibits IκB degradation and reduces phosphorylation of p65

**Fig 5:**
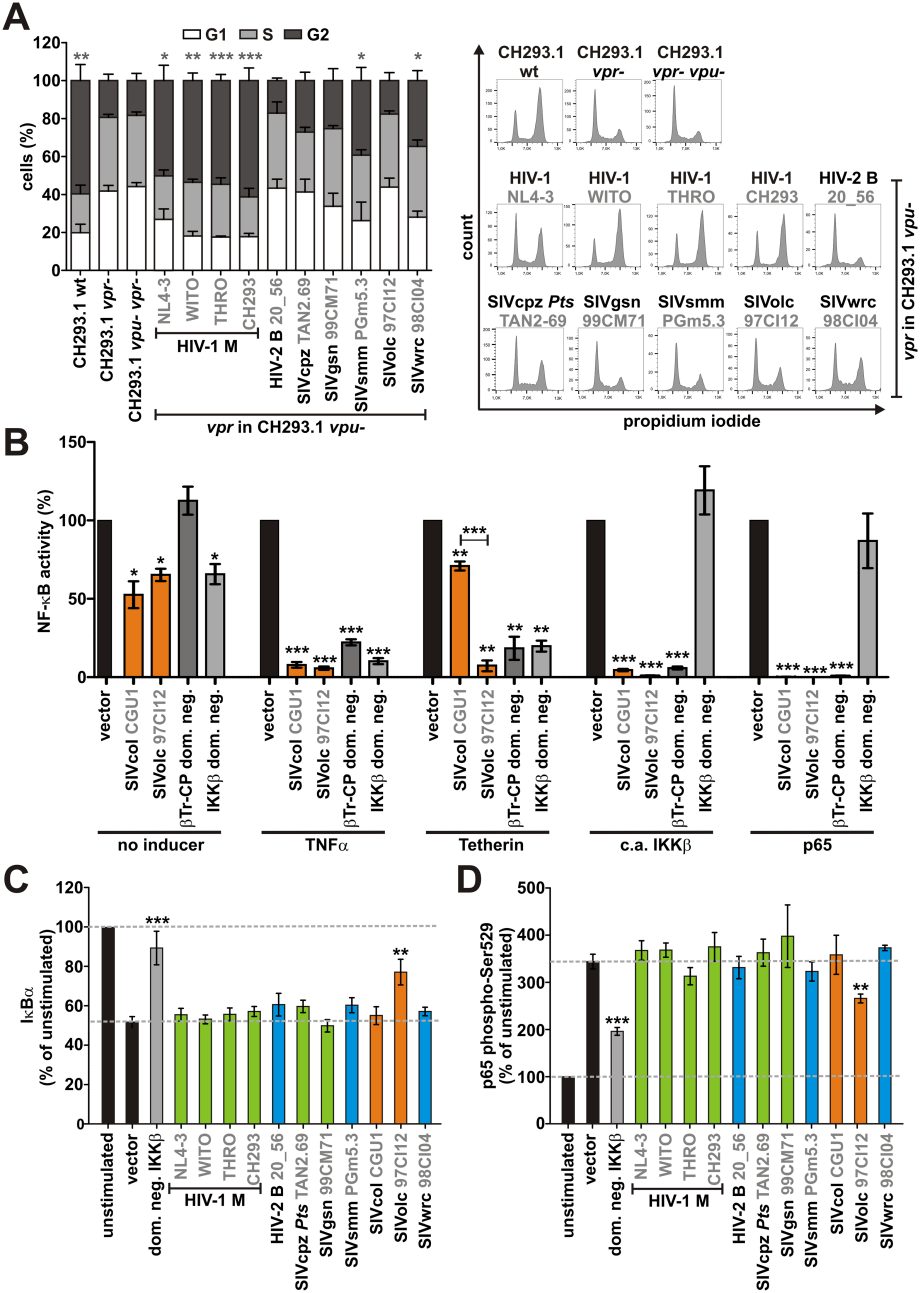
Mechanisms of SIVolc and SIVcol Vpr-mediated inhibition of NF-κB activation. (A) Jurkat cells were transduced with VSV-G pseudotyped chimeric CH293.1 viruses expressing the indicated *vpr* alleles. 48 hr post-transduction, cell cycle progression was analyzed by propidium iodide staining and flow cytometry. Mean values of three to four independent experiments ± SEM are shown on the left, examples of primary flow cytometry data are shown on the right. Asterisks indicate statistically significant differences in the percentage of cells in the G2 phase compared to the vector control (*p<0.05; **p < 0.01; ***p < 0.001). (B) HEK293T cells were transfected and analyzed as described in Fig 2. Cells were stimulated by addition of TNFα or co-transfection of expression plasmids for Tetherin, c.a. IKKβ or p65. Dominant negative mutants (dom. neg.) of β-TrCP or IKKβ served as controls. Mean values of three independent experiments in triplicates ± SEM are shown. (C) HEK293T cells were transfected with plasmids coexpressing the indicated Vpr proteins and eGFP or a dominant-negative mutant of IKKβ. Cells were stimulated 24 hr post-transfection with TNFα (10 ng/ml) or left untreated. 15 min after stimulation, cells were harvested, fixed, and permeabilized and levels of IκBα were analyzed by flow cytometry. (D) Levels of phosphorylated p65 (Ser529) were determined by flow cytometry as described for (C). Mean values of three to six independent experiments ± SEM are shown. In (B) to (D), asterisks indicate a statistically significant difference compared to the vector control (*p<0.05; **p < 0.01; ***p < 0.001).

HIV-1 Vpr is well-known for its ability to induce a G2 cell cycle arrest, a process that involves the recruitment of a DCAF1/DDB1/Cul4 E3 ligase complex and has been shown to mediate escape from innate immunity [31]. To investigate whether the inhibition of NF-κB activation by SIVolc and SIVcol Vpr depends on this activity, we infected Jurkat T cells with the chimeric CH293.1 viruses and analyzed cell cycle progression by flow cytometry. While HIV-1 Vprs inhibited the G2/M transition as expected, this was not the case for SIVolc Vpr (Fig 5A). Thus, the ability of Vpr proteins to inhibit NF-κB activation did not correlate with the induction of cell cycle arrest. In agreement with this finding, SIVcol and SIVolc efficiently suppressed the activation of NF-κB upon knockdown of DCAF1 (S5A Fig).

To further elucidate the mechanisms underlying Vpr-mediated modulation of NF-κB activation, we directly compared the ability of SIVolc and SIVcol Vpr to inhibit NF-κB activation upon TNFα stimulation or overexpression of the restriction factor Tetherin, a constitutively active mutant of IKKβ, or p65/NF-κB itself. Dominant negative mutants of IKKβ and βTr-CP, which prevent the degradation of IκB, served as controls. While SIVolc Vpr generally suppressed NF-κB activity by >90%, SIVcol Vpr reduced Tetherin-induced signaling only by about 30% (Fig 5B). These findings suggest that the distantly related SIVcol and SIVolc Vprs have evolved different mechanisms to inhibit NF-κB-mediated immune activation. In accordance with this, SIVolc, but not SIVcol stabilized IκB (Figs 5C and 3A, S5B Fig) and reduced the activating phosphorylation of p65 at serine 529 (Figs 5D and 3A, S5C Fig) upon TNFα stimulation. Thus, while both, SIVcol and SIVolc Vpr suppress NF-κB activation independently of DCAF1 and the induction of a cell cycle arrest, only SIVolc Vpr prevents degradation of IκB and phosphorylation of p65.

### NF-κB inhibition reduces SIVolc and SIVcol LTR-driven gene expression

**Fig 6:**
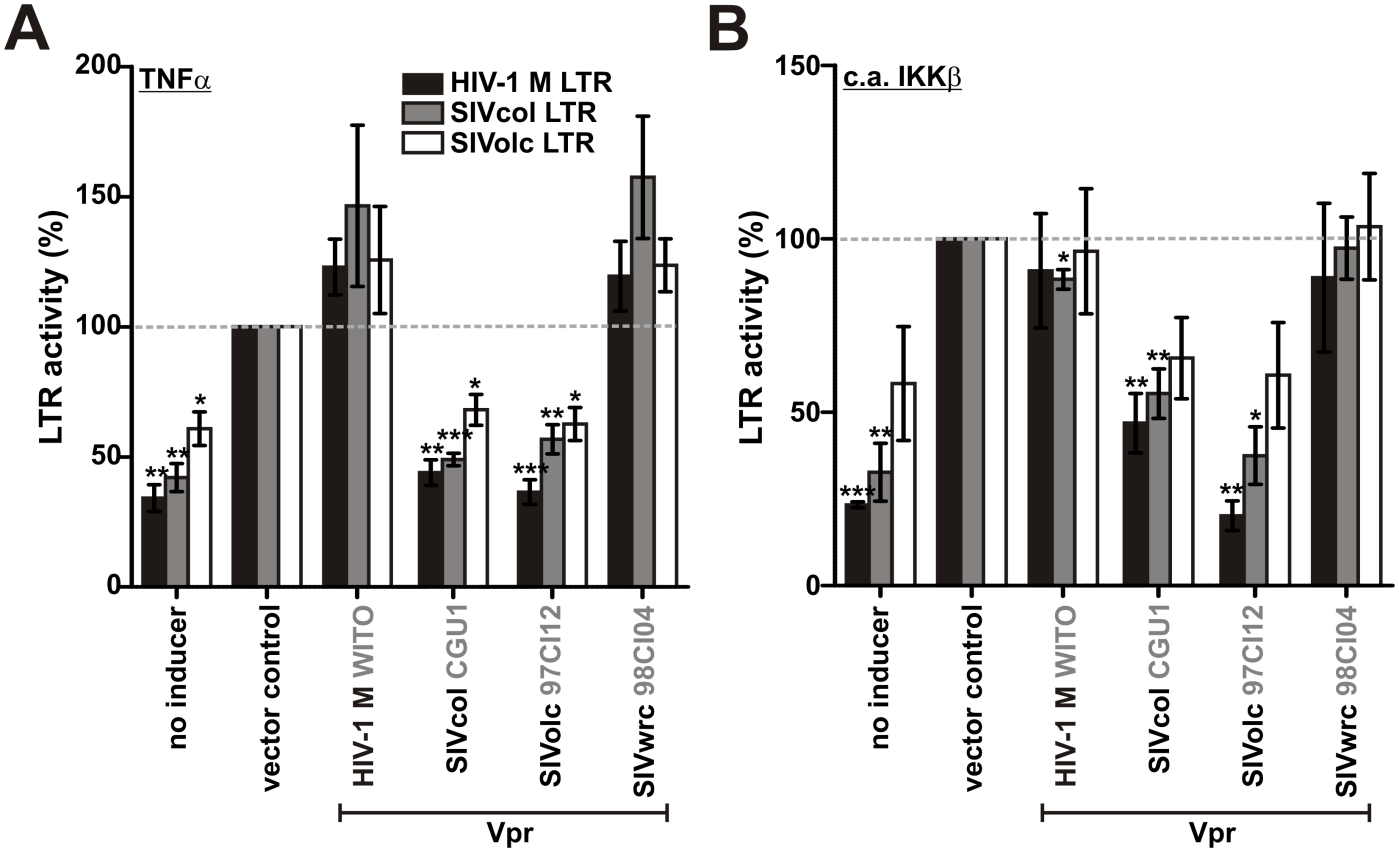
NF-κB inhibition by SIVolc and SIVcol Vpr reduces LTR-driven gene expression. (A, B) HEK293T cells were cotransfected with the indicated *vpr* alleles, a firefly luciferase reporter construct under the control of the HIV-1 M, SIVcol or SIVolc LTR promoter, and a *Gaussia* luciferase construct for normalization. Cells were (A) stimulated with TNFα or (B) cotransfected with a constitutively active mutant of IKKβ (c.a. IKKβ). Luciferase activities were determined 40 hr post-transfection. Mean values of four independent experiments in triplicates ± SEM are shown (*p<0.05; **p < 0.01; ***p < 0.001).

HIV-1 boosts NF-κB activation during early stages of its replication cycle to ensure efficient LTR-driven expression of viral genes, while it suppresses activation of this transcription factors during later stages to minimize cellular antiviral gene expression [6]. Similar to HIV-1 LTRs, those of SIVolc and SIVcol contain putative NF-κB binding sites (S6A Fig). To analyze NF-κB dependency, we amplified the LTR sequences of these simian viruses from cDNA of naturally infected monkeys and generated firefly luciferase reporter constructs. Experiments in transfected HEK293T cells revealed that these promoters are activated upon stimulation with TNFα (Fig 6A) or a constitutively active mutant of IKKβ (Fig 6B), similarly to an HIV-1 LTR promoter. LTR activity was suppressed by SIVolc and SIVcol Vpr (Fig 6A and 6B), and mutational analyses showed that this inhibitory activity depends on the presence of NF-κB, but not Sp1 binding sites (S6B Fig). In summary, our results suggest that SIVolc and SIVcol may also benefit from a temporal regulation of NF-κB activation that enables initiation of viral gene expression during early stages while minimizing cellular antiviral gene expression during late stages of the viral replication cycle.

### *De-novo* expressed but not virion-associated SIVolc Vpr inhibits NF-κB

**Fig 7:**
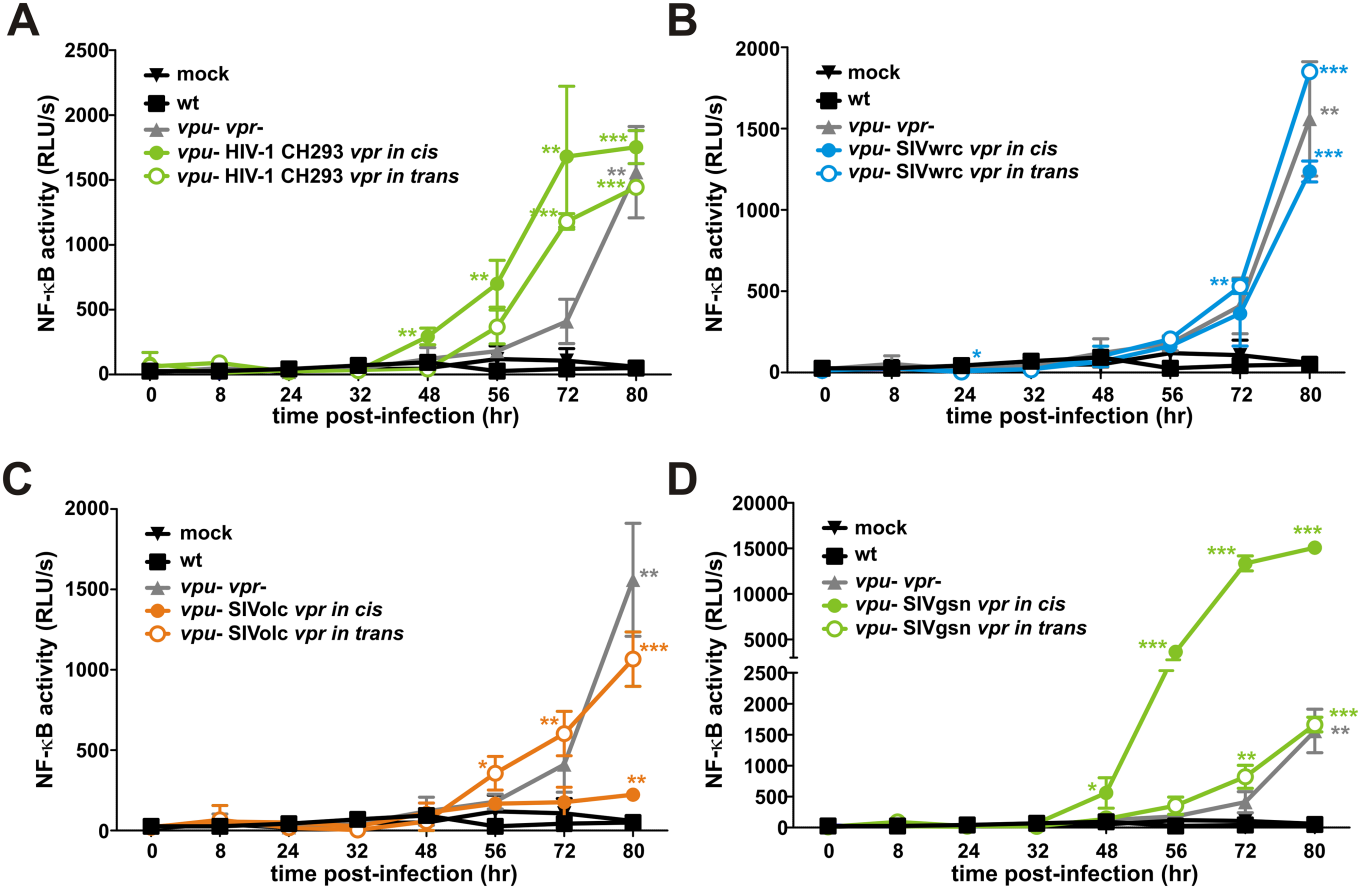
Virion-associated Vpr does not affect the activation of NF-κB in infected T cells. (A-D) SupD1 cells were transduced with the indicated VSV-G pseudotyped CH293.1 constructs. (A) HIV-1 CH293, (B) SIVwrc, (C) SIVolc, or (D) SIVgsn Vpr were either delivered *in cis* (*i*.*e*. incorporated in viral particles AND encoded in the viral genome) or *in trans* (*i*.*e*. incorporated in viral particles, but not encoded in the viral genome). CH293.1 wild type and CH293.1 *vpu*- *vpr*- served as controls. Cells were harvested at the indicated time points post-transduction to determine the activation levels of NF-κB. The mean values of triplicate infections ± SD are shown. Asterisks indicate a statistically significant difference compared to the mock control (*p<0.05; **p < 0.01; ***p < 0.001).

Vpr may act early and late on NF-κB activity because it is expressed during later stages of the replication cycle, but also incorporated into viral particles. To determine whether virion-associated and/or *de-novo* expressed Vpr accounts for the effect on NF-κB activity, HEK293T cells were transfected either with HIV-1 CH293.1 *vpu*- chimeras expressing heterologous Vpr proteins *in cis* or cotransfected with CH293.1 *vpu*- *vpr*- and an expression vector for Vpr *in trans*. In the latter case, Vpr is incorporated into virions but not produced in newly infected cells. Western blotting confirmed that most Vprs were efficiently incorporated (S7A Fig). Only SIVwrc Vpr was hardly detectable in virions if provided *in cis* (S7A Fig, Fig 4B), and the results obtained with this Vpr need to be interpreted with caution in this assay. The *in cis* or *in trans* complemented viruses were used to infect SupD1 reporter cells, and NF-κB activity was monitored for 80 hours. Infections rates were controlled by flow cytometric analysis (S7B Fig). Only *vpu*/*vpr* defective but not wild type HIV-1 CH293.1 induced the activation of NF-κB (Fig 7A, S7C Fig). Immune activation by CH293.1 *vpu*- *vpr*- could not be suppressed by *in trans* or *in cis* complementation with CH293 or SIVwrc Vpr, confirming that these proteins do not inhibit NF-κB activation (Fig 7A and 7B, S7C Fig). In contrast, expression of SIVolc Vpr *in cis* but not *in trans* suppressed activation of NF-κB by *vpu*-deficient CH293.1 constructs (Fig 7C, S7C Fig). Thus, only *de-novo* synthesized but not virion-associated SIVolc Vpr modulates the activation of NF-κB in infected T cells. Similarly, SIVgsn Vpr boosted NF-κB activation *in cis* but not *in trans* (Fig 7D, S7C Fig). Notably, the results were not biased by multiple rounds of infection as treatment with the protease inhibitor Darunavir did not significantly affect induction of NF-κB activation upon HIV-1 infection or NF-κB inhibition by SIVolc Vpr, although it efficiently blocked *de novo* generation of infectious HIV-1 particles (S7D–S7F Fig). Thus, SIVolc Vpr will not interfere with efficient initiation of viral gene expression early after infection, since *de novo* synthesis of this viral protein is required to suppress the activation of NF-κB in infected T cells.

## Discussion

**Fig 8:**
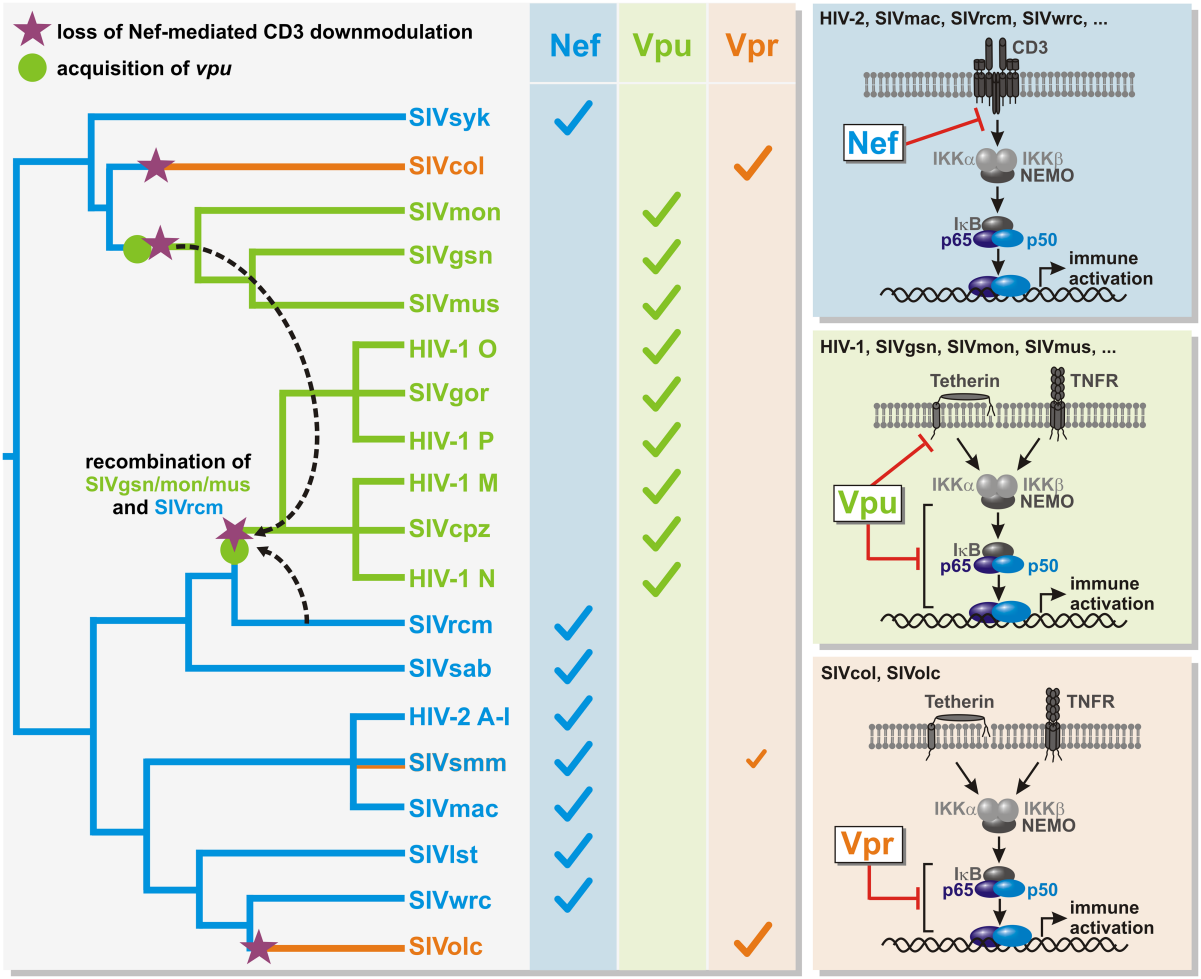
Vpu-, Vpr- and Nef-mediated inhibition of immune activation. The left panel illustrates the emergence of Vpu-, Vpr- and Nef-mediated suppression of immune activation during primate lentiviral evolution: Most primate lentiviruses use their Nef proteins to prevent T cell activation via downmodulation of CD3 (blue). HIV-1, SIVcpz/gor as well as SIVgsn/mon/mus encode a *vpu* gene and evolved Vpu-mediated inhibition of NF-κB activation (green). Nef-mediated CD3 downmodulation was lost in these viruses (violet star). In SIVcol and SIVolc, the loss of CD3 downmodulation by Nef is associated with the evolution of Vpr-mediated suppression of NF-κB signaling (orange). The tree is a schematic representation of primate lentiviral evolution and based on previous phylogenetic analyses [12]; branch lengths do not correlate with phylogenetic relationships. While Nef directly targets the CD3 receptor to prevent downstream NF-κB signaling, Vpu and SIVcol/SIVolc Vpr target the canonical NF-κB signaling cascade further downstream, independently of the receptor (right panels). Some Vpu proteins additionally counteract the host restriction factor Tetherin, which also acts as an immune sensor.

In this study, we show that primate lentiviruses use at least three alternative proteins (*i*.*e*. Nef, Vpu or Vpr) to suppress NF-κB-mediated immune activation in infected cells. HIV-2 and the majority of SIVs reduce T cell activation and NF-κB activation by Nef-mediated downmodulation of CD3 [10,11]. However, this function was lost independently twice during lentiviral evolution when the precursors of the SIVgsn/mon/mus and SIVcpz/gor/HIV-1 lineages acquired a *vpu* gene (Fig 8, left panel). We have recently shown that Vpu proteins directly target the canonical NF-κB signaling pathway and inhibit nuclear translocation of p65 to suppress expression of type I IFNs and other antiviral factors [6]. This function is highly conserved among primate lentiviruses encoding *vpu*, suggesting that the evolution of Vpu-mediated NF-κB inhibition has relieved the selection pressure for suppressing T cell activation by Nef. Here, we demonstrate that SIVcol and SIVolc, which lack a *vpu* gene and fail to efficiently downmodulate CD3 via Nef, evolved Vpr as potent inhibitor of NF-κB activity and consequently type I IFN expression.

Our findings are another example for the enormous functional plasticity of lentiviral accessory proteins and add to the accumulating evidence that these proteins often compensate for each other or cooperate to accomplish the same goal. For example, a similar evolutionary toggling between accessory viral proteins has been described for the counteraction of cellular restriction factors: While HIV-2 and related SIV strains use Vpx to antagonize the host restriction factor SAMHD1 in their respective host species, other primate lentiviruses achieve this by using Vpr [32]. Similarly, primate lentiviruses switched several times between Vpu- and Nef-mediated Tetherin antagonism before giving rise to HIV-1 [33]. Finally, three lentiviral proteins (*i*.*e*. Nef, Vpu and Env) cooperate in the downmodulation of CD4 [13].

This enormous functional divergence of accessory proteins even from the same group or lineage of HIV or SIV illustrates that results obtained with a single allele need to be interpreted with great caution. Thus, although all SIVcol and SIVolc Vprs analyzed in this study efficiently suppressed the activation of NF-κB, other isolates of these viruses may use yet another strategy to minimize immune activation. In fact, the large differences in modulation of NF-κB activity by HIV-1 Vpr proteins observed in the present study may explain why previous reports on the role of Vpr in immune activation yielded contradictory results [16,19,20,34–43]. While several groups reported reduced nuclear translocation of p65 and decreased secretion of IFNs in the presence of Vpr [16,34–38], others observed no effect on nuclear translocation and DNA binding of this transcription factor [17], or even reported increased NF-κB-mediated immune activation [19,20,39–43]. Furthermore, modulation of NF-κB activity may not only depend on the Vpr proteins analyzed but also on the stimulus used to activate NF-κB signaling. We therefore examined several stimuli (TNFα, Tetherin, IKKβ, p65, Sendai virus) in various cell types from different species (PBMCs, SupD1, HEK293T, COS-7). To better mimic the *in vivo* situation, we generated chimeric HIV-1 constructs and monitored the effects of Vpr on NF-κB and immune activation in virally infected human PBMCs. SIVolc and SIVcol Vpr potently suppressed NF-κB activation under all experimental conditions. Flow cytometric analyses revealed that SIVolc stabilizes IκBα upon TNFα stimulation. As expected, the stabilization of this endogenous NF-κB inhibitor also prevented phosphorylation of p65 at serine 529 by casein kinase II [44]. Notably, SIVcol Vpr seems to target different steps of the NF-κB signaling cascade as it neither affected p65 phosphorylation nor IκBα degradation. Moreover, SIVcol Vpr suppressed Tetherin-induced NF-κB activation by only 30%, while SIVolc Vpr inhibited it by more than 90%.

It has been suggested that Vpr may suppress NF-κB activation by preventing the sensing of reverse transcription intermediates (RTIs) [31]. Laguette and colleagues showed that HIV-1 Vpr induces the degradation of RTIs by recruitment of the SLX4-associated MUS81-EME1 endonucleases via DCAF1, a process that might also prevent G2/M cell cycle transition. A more recent study, however, suggested that some Vpr proteins can induce a cell cycle arrest also in the absence of SLX4 [45]. Here, we show that the ability of SIVcol and SIVolc Vpr to suppress NF-κB activation is independent of the induction of a cell cycle arrest and the adaptor protein DCAF1. In agreement with this, SIVcol and SIVolc Vpr inhibited NF-κB in both, human and simian cells, although they fail to recruit the human SLX4 complex and do not induce a cell cycle arrest in human cells [46].

In our experiments, expression of HIV-1 Vpu and SIVolc Vpr resulted in a 2- to 3-fold decrease in IFNβ mRNA levels in infected cells (Fig 4E). Although these differences may seem low, it is very likely that the suppression of NF-κB activation by Vpu and/or Vpr confers a selection advantage to the virus: First, the observation that Vpu-mediated NF-κB inhibition is conserved among almost all lentiviruses encoding this accessory gene suggests that this activity constitutes a significant fitness advantage. Second, NF-κB is a broad regulator of antiviral gene expression, and its modulation will probably not only affect type I IFN production, but also a variety of other antiviral proteins that cooperate in inhibiting viral replication. Finally, the inhibitory effects determined by qRT-PCR may have been masked by uninfected bystander cells and suppression of antiviral gene expression is presumably more pronounced within infected cells. Global transcriptome analyses in combination with *ex vivo* or even *in vivo* replication kinetics will be necessary to decipher the overall effects of Vpu-/Vpr-mediated NF-κB inhibition on cellular gene expression, viral replication and spread.

While it is not known whether Nef-mediated downmodulation of CD3 was lost before or after the evolution of Vpr-mediated NF-κB inhibition, it is clear that both accessory proteins target the NF-κB signaling cascade at different steps (Fig 8, right panels). By downmodulating CD3, Nef specifically inhibits the very first step of the T cell receptor signaling pathway, whereas SIVolc and SIVcol Vpr target the NF-κB signaling cascade further downstream and may thus exert inhibitory activities on several pathways that culminate in the activation of NF-κB. Although each viral particle contains about 275 Vpr molecules [47], inhibition of NF-κB signaling requires the *de-novo* synthesis of SIVolc Vpr. Thus, Vpr (like Vpu) suppresses NF-κB activity only during late stages of the viral replication cycle, thereby enabling the activation of this transcription factor during early stages to initiate LTR-dependent viral gene expression. As soon as the viral Tat protein ensures efficient viral gene expression, Vpr (or Vpu) inhibit the activation of NF-κB to limit immune activation and antiviral gene expression.

In summary, our findings highlight the key role of NF-κB in antiviral immunity and show that primate lentiviruses follow distinct evolutionary paths to balance NF-κB-dependent expression of viral and antiviral genes. The present results improve our understanding of the modulation of NF-κB-driven proinflammatory gene expression and thus shed light on the establishment of viral latency and the pathophysiology of HIV infections, since chronic immune activation is an important driver of the progression to AIDS. Finally, our data add to the accumulating evidence that lentiviral accessory proteins do not only directly target restriction factors to evade the immune system but also interfere with their expression by modulating innate signaling cascades.

## Materials and methods

### Ethical statement

Experiments involving human peripheral blood mononuclear cells were reviewed and approved by the Institutional Review Board (*i*.*e*. the Ethics Committee of Ulm University), and individuals and/or their legal guardians provided written informed consent prior to donating blood. All blood samples were anonymized before use. The use of established cell lines (HEK293T, COS-7, TZM-bl and Jurkat cells) did not require the approval of the Institutional Review Board. No non-human primates were involved, harmed, sampled or kept for this study. cDNA of mantled guerezas (*Colobus guereza*) had been obtained from bushmeat samples of wild-caught animals in previous studies [48,49]. Similarly, the cDNA of a Peter’s Angola colobus (*Colobus angolensis palliatus*) used in this study had been prepared before [50].

### Expression vectors

Primers flanking the *nef* (oligonucleotides P1, P2) and *vpr* (oligonucleotides (P11, P12) open reading frame (ORF) were used to PCR-amplify the genes from cDNA of mantled guerezas (*Colobus guereza*) infected with SIVcol CM243 or CM1437. PCR products from five independent amplifications were sequenced to identify the most abundant sequence variant for subsequent cloning. SIVcol *nef* alleles were cloned via HpaI and MluI restriction sites into HIV-1 NL4-3-based proviral constructs coexpressing eGFP via an internal ribosome entry site (IRES) [51] (oligonucleotides P3-P10). NL4-3 IRES eGFP reporter viruses containing SIVolc and SIVwrc *nef* alleles have been described before [11]. SIVcol CM243 and CM1437 *vpr* alleles were fused to an N-terminal AU-1 tag and cloned via XbaI and MluI restriction sites into bicistronic pCG vectors coexpressing eGFP via an IRES (oligonucleotides P13, P14). All remaining *vpr* alleles were either chemically synthesized or amplified from proviral constructs before cloning into the pCG expression vector. Notably, N-terminal tagging was previously shown to not affect Vpr function [31]. Human SERINC5 was expressed from PBJ6 (derived from PBJ5 by removing the SV40 origin of replication from the SV40-HTLV-1 hybrid promoter region) expression vectors [52]. The coding sequence of colobus CD3ζ was amplified essentially as described [50]. Total RNA from PBMCs of a Peter’s Angola colobus (*Colobus angolensis palliatus*) was extracted using Qiagen RNeasy Plus Mini Kit and was used to prepare cDNA using Transcriptor High Fidelity cDNA Synthesis Kit (Roche). Nested PCR was performed on cDNA using Pfx Supermix (Life Technologies) and two primer pairs binding in untranslated regions (oligonucleotides P15-P18). PCR products were sequenced and the resulting sequences were assembled to build a reference coding sequence. The PCR products were subsequently cloned into the pCR-BluntII-TOPO vector (Life Technologies). Five individual clones were sequenced using M13 fw and M13 rev primers as well as two CD3ζ specific sequencing primers (oligonucleotides P19, P20). The intracellular domain of CD3ζ was fused to the extracellular and transmembrane domain of human CD8 (amino acids 1–205), and cloned into the CMV-promoter based pCG expression vector via XbaI/MluI. Oligonucleotides P21 and P22 were used to PCR-amplify the 3’ LTR from cDNA of mantled guerezas infected with SIVcol CM243. PCR products from five independent amplifications were sequenced to identify the most abundant sequence variant for subsequent cloning. The SIVolc 97CI12 LTR sequence was derived from the Los Alamos sequence database and synthesized (Baseclear). SIVcol CM243 (oligonucleotides P23, P24) and SIVolc 97CI12 were cloned via MluI and XhoI restriction sites into pGL3-enhancer vectors expressing firefly luciferase under the control of the respective LTR. Please refer to S1, S2, S3 and S4 Tables in the supporting information files for oligonucleotide sequences P1-P24.

### HIV-1 CH293 proviral constructs

Generation of the infectious molecular clone (IMC) of the HIV-1 group M subtype C chronic control virus CH293 has been described previously [28]. To monitor the ability of Vpr proteins to modulate NF-κB activation during infection, we created a variant of CH293 allowing the insertion of AU1-tagged *vpr* alleles from various HIV and SIV species via unique XbaI and MluI restriction sites. This modified version was named CH293.1. The CH293 *vpr* open reading frame (ORF) in the *vif/tat1* intergenic region was replaced by XbaI and MluI restriction sites and the following additional mutations were introduced using overlap extension PCR: The *vpr* start codon in *vif* was mutated with a synonymous mutation in the *vif* ORF, so that the Vif protein would remain unchanged and functional. Since the insertion of a new *vpr* gene also introduces a second *tat* start codon, an additional base (cytosine) was introduced downstream of the MluI restriction site, prior to the original *tat* start codon. This creates a frameshift resulting in premature stop codons in all possible reading frames of the inserted second *tat* start codon to avoid the expression of an additional Tat protein (oligonucleotides P25-P28). Two additional XbaI restriction sites within the *env* sequence of CH293 were mutated with synonymous mutations in the *env* ORF to facilitate cloning via XbaI/MluI (oligonucleotides P29- P32). Finally, v*pr* alleles with N-terminal AU1 tag were excised from pCG expression constructs and cloned into CH293.1 via the XbaI/MluI restriction sites. When indicated, Vpu expression was disrupted by insertion of two stop codons downstream of the *vpu* start codon using the QuikChange II XL site-directed mutagenesis kit (Agilent Technologies) according to the manufacturer’s instructions (oligonucleotides P33, P34). Please refer to S5 Table of the supporting information files for oligonucleotide sequences P25-P34.

### Cell culture

Human Embryonic Kidney (HEK) 293T cells and COS-7 cells (both obtained from the American Type Culture Collection (ATCC)) were first described by DuBridge *et al*. [53] and Gluzman [54], respectively. TZM-bl reporter cells (kindly provided by Drs. Kappes and Wu and Tranzyme Inc. through the NIH AIDS Reagent Program [55]) were used to determine infectious virus yield. All three cell lines were maintained in Dulbecco’s modified Eagle medium (DMEM) supplemented with 10% fetal calf serum (FCS), 2 mM glutamine, 100 μg/ml streptomycin and 100 U/ml penicillin. Jurkat cells (obtained from the American Type Culture Collection (ATCC)) were generated by Schneider *et al*. [56] and cultivated in RPMI 1640 medium supplemented with 10% FCS, 2 mM glutamine, streptomycin (100 μg/ml) and penicillin (100 U/ml). The SupD1 cell line (generated in our lab [6]) expresses a short-lived version of an NF-κB-dependent firefly luciferase reporter. SupD1 cells were cultivated in RPMI 1640 medium supplemented with 10% FCS, 2 mM glutamine, streptomycin (100 μg/ml), penicillin (100 U/ml) and hygromycin B (200 μg/ml). Peripheral blood mononuclear cells (PBMCs) from healthy human donors were isolated from buffy coats using lymphocyte separation medium (Biocoll separating solution; Biochrom) and cultivated in RPMI 1640 medium supplemented with 10% FCS, 2 mM glutamine, streptomycin (100 μg/ml), penicillin (100 U/ml), IL-2 (10 ng/ml) (Miltenyi Biotec) and PHA (1 μg/ml) (Thermo Scientific).

### NF-κB reporter assay in HEK293T and COS-7 cells

To determine the effect of Vpr on NF-κB activity, HEK293T cells were seeded in 96-well plates coated with poly-L-lysine and transfected in triplicates using a standard calcium phosphate transfection protocol [10]. COS-7 cells were seeded in non-coated 96-well plates and transfected using polyethylenimine (PEI). Cells were cotransfected with a firefly luciferase reporter construct under the control of three NF-κB binding sites (100 ng), a *Gaussia* luciferase construct under the control of a constitutively active pTAL promoter (5 ng) for normalization, and expression vectors for different Vpr proteins (100 ng) or CH293.1 proviral constructs expressing heterologous Vpr proteins (100 ng). To determine the influence of DCAF1 on modulation of NF-κB activation by Vpr, DCAF1 was depleted by cotransfecting expression vectors for three different shRNAs (50 ng) [57]. To activate NF-κB, Tetherin (40 ng), p65 (1.6 ng) or a constitutively active mutant of IKKβ (40 ng) was cotransfected or cells were stimulated with TNFα (20 ng/ml) for 24 hr. 40 hr post-transfection, a dual luciferase assay was performed and the firefly luciferase signals were normalized to the corresponding *Gaussia* luciferase control values.

### IFNβ-promoter reporter assay in HEK293T cells

To determine the effect of Vpr proteins on IFNβ-promoter activity, dual luciferase assays were performed as described above. Cells were transfected with firefly luciferase reporter constructs under the control of the wild type IFNβ-promoter (100 ng) or a mutant thereof lacking the NF-κB binding site as previously described [6]. The promoter was activated by stimulation with Sendai virus for 24 hr.

### LTR reporter assay in HEK293T cells

To determine the effect of Vpr proteins on LTR activity, reporter constructs were generated in which a firefly luciferase reporter gene is expressed under the control of the SIVcol CM243, SIVolc 97CI12 and HIV-1 NL4-3 LTR, or mutants thereof with mutated NF-κB or Sp1 binding sites. HEK293T cells were cotransfected with these constructs (100 ng), expression vectors for different Vpr proteins (100 ng) and *Gaussia* luciferase vectors under the control of a constitutively active pTAL promoter (5 ng) for normalization. LTR-mediated transcription was activated by cotransfection of a constitutively active mutant of IKKβ (40 ng), Sp1 (40 ng) or by stimulation with TNFα (20 ng/ml). 40 hr post-transfection, a dual luciferase assay was performed and the firefly luciferase signals were normalized to the corresponding *Gaussia* luciferase control values.

### Virus production

To generate virus stocks, HEK293T cells were transfected in 6-well plates with proviral HIV-1 constructs (5 μg) using a standard calcium phosphate transfection protocol [10]. For the production of VSV-G pseudotyped HIV-1 particles, the proviral constructs were cotransfected with expression plasmids for VSV-G (1 μg). For mock infection controls, HEK293T cells were treated with transfection reagents only. Supernatants were harvested 40 hr post-transfection.

### Infectivity assay

Infectious HIV-1 yield was determined by a 96-well infection assay using TZM-bl indicator cells. Briefly, 6,000 cells were seeded in 96-well plates and infected in triplicates with cell culture supernatants. Three days later, infection rates were measured using a galactosidase screen kit (GalScreen-Applied Bioscience) according to the manufacturer’s instructions. β-galactosidase activities were quantified as relative light units per second (RLU/s) using an Orion Microplate Luminometer.

### SERINC5 counteraction assay

To show expression of functional Nef proteins, HEK293T cells were cotransfected in 6-well plates with increasing doses (0; 0.1; 0.5; 2.5 μg) of SERINC5 expression vectors and HIV-1 NL4-3 proviral constructs expressing different Nef proteins (2.5 μg). 40 hr post-transfection, infectious virus yield was determined by infection of TZM-bl indicator cells for 72 hr in triplicates.

### Flow cytometry

To determine the effect of Vpr on the levels of IκBα and phosphorylation of p65 at Ser529, HEK293T cells were transfected in 6-well plates with expression vectors for different Vpr proteins (2.5 μg) coexpressing GFP via an IRES. 24 hours post-transfection cells were left untreated or stimulated with TNFα (10 ng/ml) for 15 minutes at 37°C before fixation with 4% PFA for 10 minutes at 37°C. Cells were permeabilized with ice-cold methanol for 30 minutes on ice and washed twice before staining with AF647-coupled antibodies for IκBα (BD #560817) or p65 phosphorylated at Ser529 (BD #558422) or the respective isotype controls. For analysis, the APC signals of the isotype controls were subtracted and the APC signals of the GFP positive population of the TNFα-stimulated samples were normalized to the APC signals of the GFP positive population of the corresponding unstimulated controls. To determine the ability of human and simian Nef proteins to downmodulate human CD3 (BD #555333), CXCR4 (BD #555974) or CD28 (BD #559770) from the cell surface, PBMCs were transduced with VSV-G pseudotyped HIV-1 NL4-3 expressing different Nef proteins and coexpressing GFP via an IRES. Protein surface expression was determined 3 days post-transduction. To analyze the effects of Nef on human and colobus CD3, HEK293T cells were cotransfected with proviral constructs for HIV-1 NL4-3 expressing different Nef proteins and coexpressing GFP via an IRES and constructs expressing fusion proteins between the cytoplasmic part of human or colobus CD3ζ and the extracellular part of CD8. CD8 surface expression (BD #555369) was determined 40 hr post-transfection. Infection rates of SupD1 cells or PBMCs were controlled by intracellular staining for p24 (KC57-RD1; Beckman Coulter) after fixation and permeabilization of the cells using the FIX&PERM kit (Nordic MUbio) according to the manufacturer’s instructions. Flow cytometric measurements were performed using a BD FACS Canto II flow cytometer.

### Cell cycle analysis

Jurkat cells were transduced with VSV-G pseudotyped HIV-1 CH293.1 expressing heterologous Vprs. After 48 hr, cells were fixed with 1% PFA for 20 min at RT and subsequently permeablized with 0.1% Triton to stain p24 (KC57-FITC; Beckman Coulter) for 45 min at 4°C. DNA staining was performed with propidium iodide (50 mg/ml) and 100 μg/ml RNase in 0.1% Triton for 1 hr at RT before flow cytometric analysis to determine the effect of Vpr on cell cycle phases in infected (p24+) cells.

### Apoptosis assay

HEK293T cells were transfected with pCG vectors coexpressing Vpr and eGFP. Two days post transfection, cells were stained with Fixable Viability Stain (FVS, BD #562247) and Annexin V (AnnV; BD #550474) and analyzed by flow cytometry. Cells were categorized as FVS+/AnnV+ (dead), FVS-/AnnV- (live) or FVS-/AnnV+ (early apoptosis).

### NF-κB reporter assay in HIV-1 infected cells

To determine the effect of Vpr proteins on NF-κB activity, SupD1 cells were seeded in 96-well plates and transduced in triplicates with VSV-G pseudotyped HIV-1 CH293.1 expressing heterologous Vprs. Cells were harvested at various time points post-transduction to determine NF-κB-dependent firefly luciferase activity. Transduction efficiency was monitored by flow cytometry. To determine the effect of multiple rounds of infection on NF-κB activation, SupD1 cells were infected in the presence or absence of the protease inhibitor Darunavir (10 mM). NF-κB activation was measured at various time points post-transduction and supernatants were used to infect TZM-bl indicator cells to check whether Darunavir treatment was efficient.

### Western blot

Cells were lysed in Western blot lysis buffer (150 mM NaCl, 50 mM HEPES, 5 mM EDTA, 0.1% NP40, 500 μM Na_3_VO_4_, 500 μM NaF, pH 7.5). Cell-free virions were pelleted by centrifugation of cell culture supernatants through a 20% sucrose cushion at 20,800 g for 90 min at 4°C and lysed in Western blot lysis buffer. Lysates were mixed with Protein Sample Loading Buffer (LI-COR) supplemented with 10% β-mercaptoethanol, heated at 95°C for 5 min, separated on NuPAGE 4–12% Bis-Tris Gels (Invitrogen) and blotted onto Immobilon-FL PVDF membranes (Merck Millipore). Proteins were stained using primary antibodies directed against HIV-1 Env (obtained through the NIH AIDS Reagent Program, Division of AIDS, NIAID, NIH: 16H3 mAb from Drs. Barton F. Haynes and Hua-Xin Liao) [58], p24 (Abcam #ab9071), AU-1 (Novus Biologicals #NB600-453), β-actin (Abcam #ab8226), GAPDH (BioLegend #631401), GFP (Abcam #ab290), DCAF1 (Proteintech #11612-1-AP) and Infrared Dye labeled secondary antibodies (LI-COR IRDye). Proteins were detected using an LI-COR Odyssey scanner and band intensities were quantified using LI-COR Image Studio Lite Version 3.1.

### qRT-PCR

Total RNA was isolated and purified from transduced PBMCs using the RNeasy Plus Mini Kit (QIAGEN) and residual genomic DNA was removed using the DNA-free^™^ Kit (Life Technologies #AM1906). 150 ng of RNA were reversely transcribed using the PrimeScript RT reagent Kit (TAKARA #RR037A) with oligo dT and random hexamer primers. To control complete removal of genomic DNA, control samples without reverse transcriptase were included in the reaction. Generated cDNA was subjected to quantitative real time PCR using TaqMan primer/probe sets for human IFNB1 (Thermo Fisher Scientific #Hs01077958_s1), IFI44 (Thermo Fisher Scientific #Hs00197427_m1) and GAPDH (Thermo Fisher Scientific #4310884E) as control. Ct data was processed relative to the GAPDH control.

### Statistical analysis

Statistical analyses were performed using GraphPad Prism 5.0. Two-tailed, unpaired or paired student’s t-test and one sample t-test were used to determine statistically significant differences (* p ≤ 0.05; ** p ≤ 0.01; *** p ≤ 0.001). Correlation analyses were performed using Spearman’s non-parametric correlation test.

## Supporting information

S1 Fig Protein sequences and receptor downmodulation activity of SIVcol and SIVolc Nef. (A) Sequence alignment of the Nef proteins analyzed in Fig 1. Dots indicate identical amino acids. Gaps that were introduced to improve the alignment are indicated by dashes. Functional motifs are highlighted in color (bdg., binding; AP, adaptor proteins; SH3, Src-homology 3; PAK1/2, p21 activated kinase 1; V1H, subunit H of the vacuolar membrane ATPase). (B), (C) PBMCs were transduced with VSV-G pseudotyped NL4-3 constructs coexpressing the indicated Nef proteins and eGFP via an IRES. 72 hr post-transduction, CD28 (B) or CXCR4 (C) surface levels were quantified by flow cytometry. Mean values of five infections ± SD are shown (**p < 0.01; ***p < 0.001). (D) HEK293T cells were cotransfected with HIV-1 NL4-3 IRES eGFP constructs expressing the indicated *nef* alleles and plasmids expressing fusion proteins that consist of the extracellular and transmembrane domain of human CD8 and the intracellular part of either human or colobus CD3ζ. 40 hr post transfection, CD8-CD3ζ surface expression levels were analyzed by flow cytometry. Primary FACS data of one representative experiment are shown. Numbers indicate the mean fluorescence intensities of CD8-CD3ζ APC in the eGFP negative and positive populations.
(TIF)

S2 Fig Sequences, expression, induction of apoptosis and NF-κB modulation by primate lentiviral Vpr proteins. (A) Sequence alignment of the 32 Vpr proteins analyzed in this study. Dots indicate identical amino acids. Gaps that were introduced to improve the alignment are indicated by dashes. Yellow boxes highlight conserved amino acid residues in the first α-helix, which has previously been shown to be involved in G2 arrest, nuclear localization and virion-packaging of Vpr. (B) Western blot analysis of HEK293T cells transfected with expression vectors for the indicated AU1-tagged *vpr* alleles coexpressing enhanced green fluorescent protein (eGFP) via an internal ribosomal entry site (IRES). Expression of Vpr was visualized with an antibody against the AU1-tag. eGFP and GAPDH were detected to control for transfection efficiencies and protein amounts, respectively. (C) Flow cytometric analysis of HEK293T cells transfected with the indicated Vpr expression plasmids. Viability of the cells was determined 48 hr post-transfection by staining with Annexin V and Fixable Viability Stain. Mean values of three experiments ± SEM are shown. Overexpression of the pro-apoptotic protein APOL6 [59] served as positive control. Asterisks indicate statistically significant differences in the percentage of dead cells compared to the vector control (**p < 0.01). (D) Correlation of TNFα- and IKKβ-induced NF-κB activation shown in Fig 2 (green: Vprs from lentiviruses encoding *vpu*; blue: Vprs from lentiviruses downmodulating CD3 via Nef; orange: SIVcol and SIVolc Vpr). Spearman’s non-parametric correlation coefficient (r) was calculated. (E, F) COS-7 cells (derived from African green monkeys) were cotransfected with the indicated *vpr* alleles, a firefly luciferase reporter construct under the control of three NF-κB binding sites, and a *Gaussia* luciferase construct for normalization. To activate NF-κB, cells were (E) stimulated with TNFα or (F) cotransfected with a constitutively active mutant of IKKβ (c.a. IKKβ). Luciferase activities were determined 40 hr post-transfection. Mean values of three independent experiments in triplicates ± SEM are shown. Asterisks indicate statistically significant differences compared to the vector control (**p < 0.01; ***p < 0.001).
(TIF)

S3 Fig Inhibition of IFNβ promoter activity by SIVcol and SIVolc Vpr. HEK293T cells were cotransfected with the indicated *vpr* alleles, a *Gaussia* luciferase construct for normalization, and a firefly luciferase reporter construct to determine IFNβ promoter activity (with wild type or mutated NF-κB binding site). To activate the IFNβ promoter, cells were stimulated with Sendai virus. Luciferase activities were determined 40 hr post-transfection. Mean values of three independent experiments in triplicates ± SEM are shown.
(TIF)

S4 Fig Infection rates of HIV-1 CH293.1 expressing heterologous *vpr* alleles. (A) TZM-bl reporter cells were infected with chimeric CH293.1 viruses expressing the indicated *vpr* alleles. Virus stocks were produced in HEK293T cells and pseudotyped with the glycoprotein of the vesicular stomatitis virus (VSV-G) if indicated. Three days post infection, β-galactosidase activity was determined. Mean values of three experiments with triplicate infections ± SEM are shown. (B) Mean cumulative NF-κB activity of the kinetics shown in Fig 4D was calculated. The mean values of triplicate infections ± SD are shown. Asterisks indicate significant differences compared to CH293.1 *vpu*- *vpr*- (**p < 0.01; ***p < 0.001). (C) SupD1 cells were transduced with the indicated VSV-G pseudotyped CH293.1 chimeras. 30 hr post-transduction, the percentage of p24-expressing cells was determined by flow cytometry. Values represent infection rates of the experiment shown in Fig 4D. (D) The GAPDH and IFNβ amplification products of the qRT-PCR analyses shown in Fig 4E of donor B were analyzed by gel electrophoresis. (E) PBMCs were transduced with VSV-G pseudotyped CH293.1 chimeras expressing the indicated *vpr* alleles. Cells were harvested 72 hr post-transduction, and total cellular RNA was isolated and reversely transcribed. IFI44 mRNA levels were determined by quantitative RT-PCR and normalized to GAPDH mRNA. The mean values ± SEM are shown. Asterisks indicate statistically significant differences compared to CH293.1 wild type infected cells (*p<0.05). (F) The percentage of p24-expressing cells of the experiments shown in Fig 4E and S4E Fig was determined by flow cytometry, 72 hr post-transduction. The results of three donors are shown. Donors A-C in Fig 4E, S4E and S4F Fig are identical.
(TIF)

S5 Fig Role of DCAF1 and modulation of IκBα degradation and p65 phosphorylation by Vpr. (A) HEK293T cells were cotransfected with the indicated *vpr* alleles, a firefly luciferase reporter construct under the control of three NF-κB binding sites, a *Gaussia* luciferase construct for normalization and shRNA vectors to deplete DCAF1. Cells were stimulated with TNFα (left panel) or cotransfected with a constitutively active mutant of IKKβ (c.a. IKKβ) (middle panel). Luciferase activities were determined 40 hr post-transfection. Mean values of two independent experiments performed in triplicate transfections ± SEM are shown. To confirm DCAF1 knockdown, Western blotting of transfected (i.e. eGFP positive cells) was performed 40 hr post-transfection (right panel). GAPDH served as loading control and was used to calculate relative DCAF1 levels. (B) HEK293T cells were transfected with plasmids coexpressing the indicated *vpr* alleles and eGFP or a dominant-negative mutant of IKKβ (d. n. IKKβ). Cells were stimulated 24 hr post-transfection with TNFα (10 ng/ml) or left untreated. Fifteen minutes after stimulation, cells were harvested, fixed, and permeabilized and levels of IκBα were analyzed by flow cytometry. Primary FACS data of one representative experiment are shown. Numbers indicate the mean fluorescence intensities of IκBα-APC in the eGFP negative and positive populations. (C) Levels of phosphorylated p65 (Ser529) were determined by flow cytometry as described in (B). Primary FACS data of one representative experiment are shown. Numbers indicate the mean fluorescence intensities of p65 phospho-Ser529-APC in the eGFP negative and positive populations.
(TIF)

S6 Fig Modulation of NF-κB- and Sp1-driven LTR activation by Vpr. (A) Nucleotide sequence alignment of the HIV-1 NL4-3, SIVcol CM243 and SIVolc 97CI12 LTR sequences analyzed in Fig 6A and 6B. Classical NF-κB binding sites (GGGRNNYYCC) are indicated by blue boxes [60], extended NF-κB binding sites (RGGRNNHHYYB) including sequences bound by RELA homodimers are highlighted in grey [61]. (B) HEK293T cells were cotransfected with the indicated *vpr* alleles, a firefly luciferase reporter construct under the control of the HIV-1 M LTR promoter, and a *Gaussia* luciferase construct for normalization. The LTR promoter was either intact (wt) or lacked functional NF-κB or Sp1 binding sites (NF-κB mut. and Sp1 mut., respectively). Cells were stimulated by cotransfection of a constitutively active mutant of IKKβ (c.a. IKKβ) or Sp1. Luciferase activities were determined 40 hr post-transfection. Mean values of three independent experiments in triplicates ± SEM are shown (*p<0.05; **p < 0.01).
(TIF)

S7 Fig Vpr *in trans* and *in cis* complementation of HIV-1 CH293.1 *vpu- vpr-*. (A) HEK293T cells were transfected with the indicated infectious molecular clones. The indicated *vpr* alleles were either encoded in the viral genome (*in cis*) or expressed from a pCG expression vector (*in trans*) coexpressing eGFP. If Vpr was expressed *in cis*, a vector expressing only eGFP was cotransfected. 40 hr post-transfection, cells and supernatants were harvested and Western blotting was performed to detect AU1-tagged Vpr and Gag. eGFP and β-actin were detected to control for transfection efficiencies and protein loading, respectively and served as purity controls in the supernatant. (B) SupD1 cells were transduced with the indicated VSV-G pseudotyped CH293.1 viruses expressing Vpr *in cis* or *in trans*. 30 hr post-transduction, the percentage of p24-expressing cells was determined by flow cytometry. Values represent infection rates of the experiment shown in Fig 7. (C) Mean cumulative NF-κB activity of the kinetics shown in Fig 7 was calculated. The mean values of triplicate infections ± SD are shown. Asterisks indicate significant differences compared to CH293.1 *vpu*- *vpr*- (*p<0.05; **p < 0.01; ***p < 0.001). (D) SupD1 cells were transduced with the indicated VSV-G pseudotyped CH293.1 mutants in the presence or absence of the protease inhibitor Darunavir (100 nM). As a reporter for NF-κB activation, firefly luciferase activities in the cells were determined 48 and 72 hr after transduction. The mean values of triplicate infections ± SD are shown. (E) Infection rates of the cells described in (D) were determined 48 hr after infection by p24 staining in permeabilized cells, followed by FACS analysis. (F) Supernatants of the SupD1 cells described in (D) were harvested 48 and 72 hr after infection and infectious virus yield was determined by infection of TZM-bl cells for 72 h in the presence of Darunavir (100 nM). In (D), (E) and (F), the results of the same representative experiment are shown.
(TIF)

S1 Table Oligonucleotides used to generate HIV-1 NL4-3 IRES eGFP constructs expressing different *nef* alleles. (DOCX)

S2 Table Oligonucleotides used to generate pCG IRES eGFP constructs expressing different *vpr* alleles. (DOCX)

S3 Table Oligonucleotides used to generate CD8-CD3ζ fusion constructs. (DOCX)

S4 Table Oligonucleotides used to generate pGL3 LTR firefly luciferase reporter constructs. (DOCX)

S5 Table Oligonucleotides used to generate CH293.1 proviruses expressing heterologous *vpr* alleles. (DOCX)
