# Supplementary figures and images for: Primate lentiviruses use at least three alternative strategies to suppress NF-κB-mediated immune activation
Source: PLoS Pathog. 2017 Aug 31;13(8):e1006598. doi: 10.1371/journal.ppat.1006598 (PMC5597281; doi:10.1371/journal.ppat.1006598)

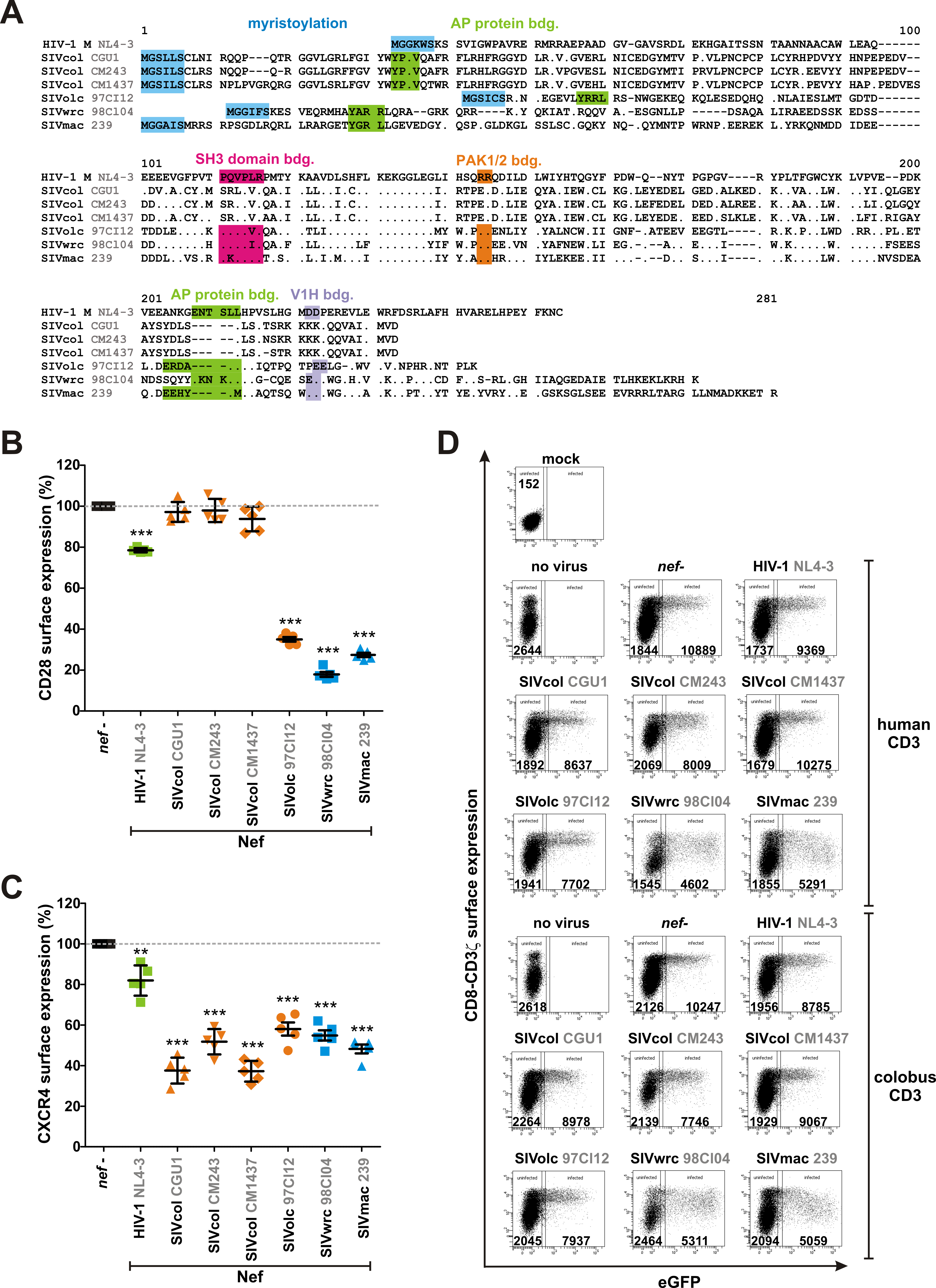

Supplement: S1 Fig — (A) Sequence alignment of the Nef proteins analyzed in Fig 1. Dots indicate identical amino acids. Gaps that were introduced to improve the alignment are indicated by dashes. Functional motifs are highlighted in color (bdg., binding; AP, adaptor proteins; SH3, Src-homology 3; PAK1/2, p21 activated kinase 1; V1H, subunit H of the vacuolar membrane ATPase). (B), (C) PBMCs were transduced with VSV-G pseudotyped NL4-3 constructs coexpressing the indicated Nef proteins and eGFP via an IRES. 72 hr post-transduction, CD28 (B) or CXCR4 (C) surface levels were quantified by flow cytometry. Mean values of five infections ± SD are shown (**p < 0.01; ***p < 0.001). (D) HEK293T cells were cotransfected with HIV-1 NL4-3 IRES eGFP constructs expressing the indicated nef alleles and plasmids expressing fusion proteins that consist of the extracellular and transmembrane domain of human CD8 and the intracellular part of either human or colobus CD3ζ. 40 hr post transfection, CD8-CD3ζ surface expression levels were analyzed by flow cytometry. Primary FACS data of one representative experiment are shown. Numbers indicate the mean fluorescence intensities of CD8-CD3ζ APC in the eGFP negative and positive populations. (TIF) [file ppat.1006598.s001.tif]

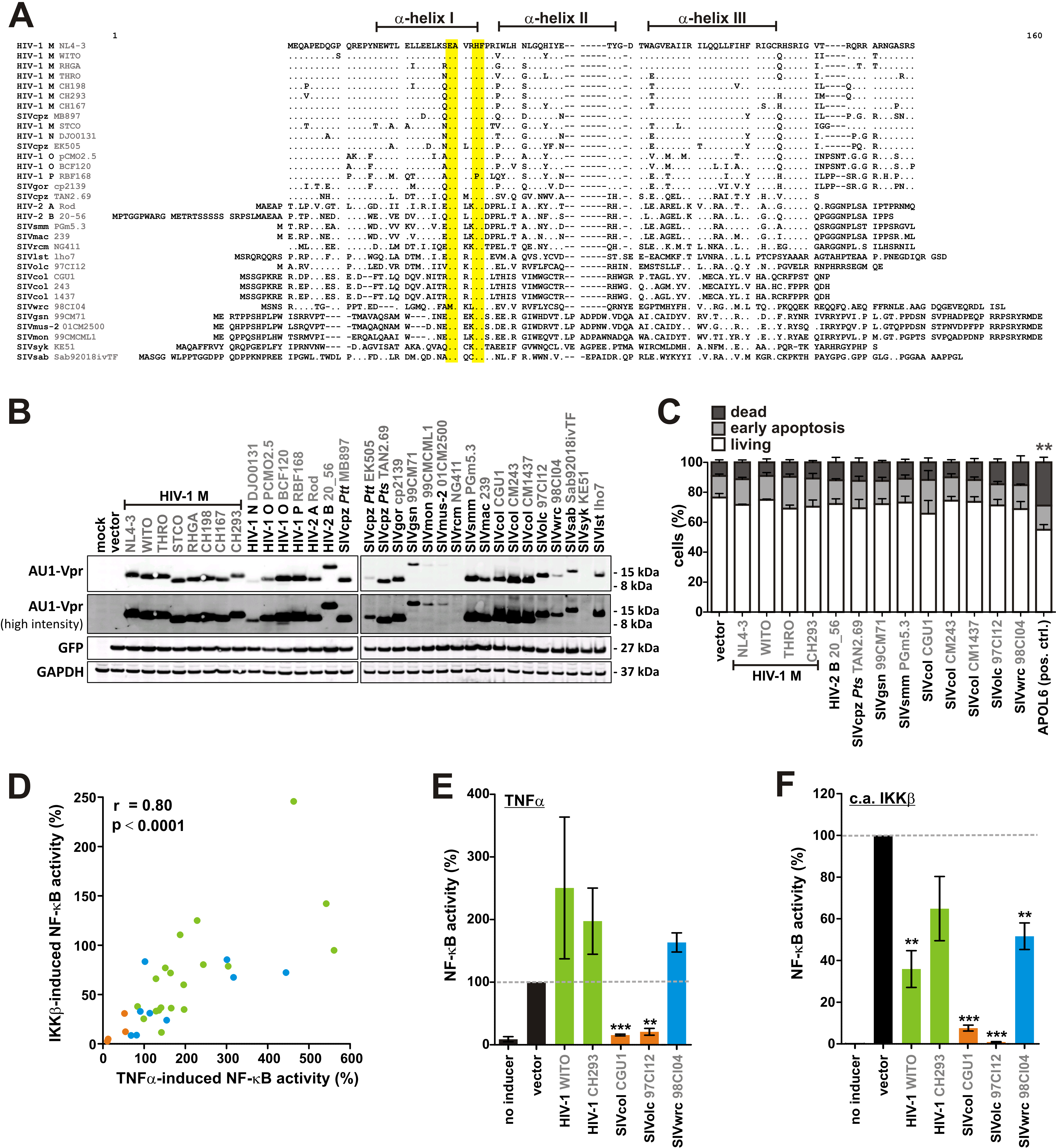

Supplement: S2 Fig — (A) Sequence alignment of the 32 Vpr proteins analyzed in this study. Dots indicate identical amino acids. Gaps that were introduced to improve the alignment are indicated by dashes. Yellow boxes highlight conserved amino acid residues in the first α-helix, which has previously been shown to be involved in G2 arrest, nuclear localization and virion-packaging of Vpr. (B) Western blot analysis of HEK293T cells transfected with expression vectors for the indicated AU1-tagged vpr alleles coexpressing enhanced green fluorescent protein (eGFP) via an internal ribosomal entry site (IRES). Expression of Vpr was visualized with an antibody against the AU1-tag. eGFP and GAPDH were detected to control for transfection efficiencies and protein amounts, respectively. (C) Flow cytometric analysis of HEK293T cells transfected with the indicated Vpr expression plasmids. Viability of the cells was determined 48 hr post-transfection by staining with Annexin V and Fixable Viability Stain. Mean values of three experiments ± SEM are shown. Overexpression of the pro-apoptotic protein APOL6 [59] served as positive control. Asterisks indicate statistically significant differences in the percentage of dead cells compared to the vector control (**p < 0.01). (D) Correlation of TNFα- and IKKβ-induced NF-κB activation shown in Fig 2 (green: Vprs from lentiviruses encoding vpu; blue: Vprs from lentiviruses downmodulating CD3 via Nef; orange: SIVcol and SIVolc Vpr). Spearman’s non-parametric correlation coefficient (r) was calculated. (E, F) COS-7 cells (derived from African green monkeys) were cotransfected with the indicated vpr alleles, a firefly luciferase reporter construct under the control of three NF-κB binding sites, and a Gaussia luciferase construct for normalization. To activate NF-κB, cells were (E) stimulated with TNFα or (F) cotransfected with a constitutively active mutant of IKKβ (c.a. IKKβ). Luciferase activities were determined 40 hr post-transfection. Mean values of three inde [file ppat.1006598.s002.tif]

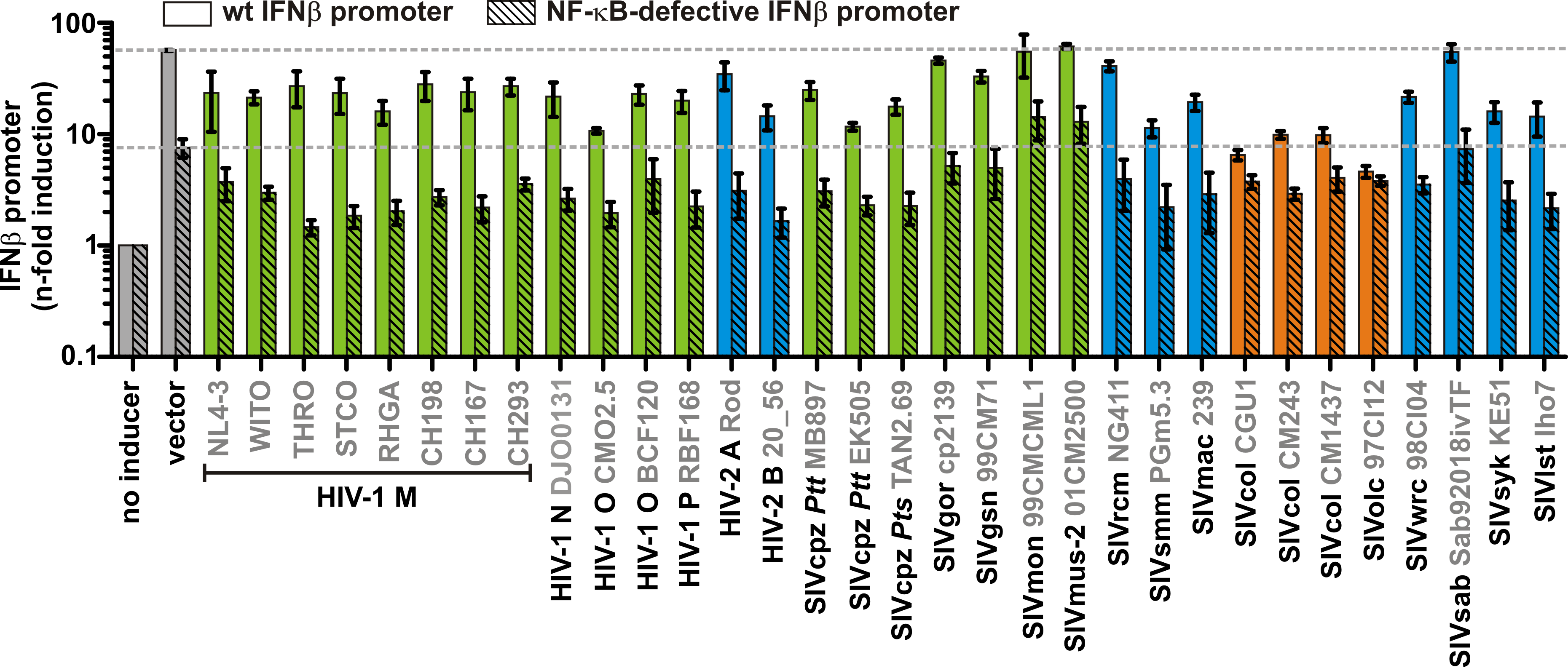

Supplement: S3 Fig — HEK293T cells were cotransfected with the indicated vpr alleles, a Gaussia luciferase construct for normalization, and a firefly luciferase reporter construct to determine IFNβ promoter activity (with wild type or mutated NF-κB binding site). To activate the IFNβ promoter, cells were stimulated with Sendai virus. Luciferase activities were determined 40 hr post-transfection. Mean values of three independent experiments in triplicates ± SEM are shown. (TIF) [file ppat.1006598.s003.tif]

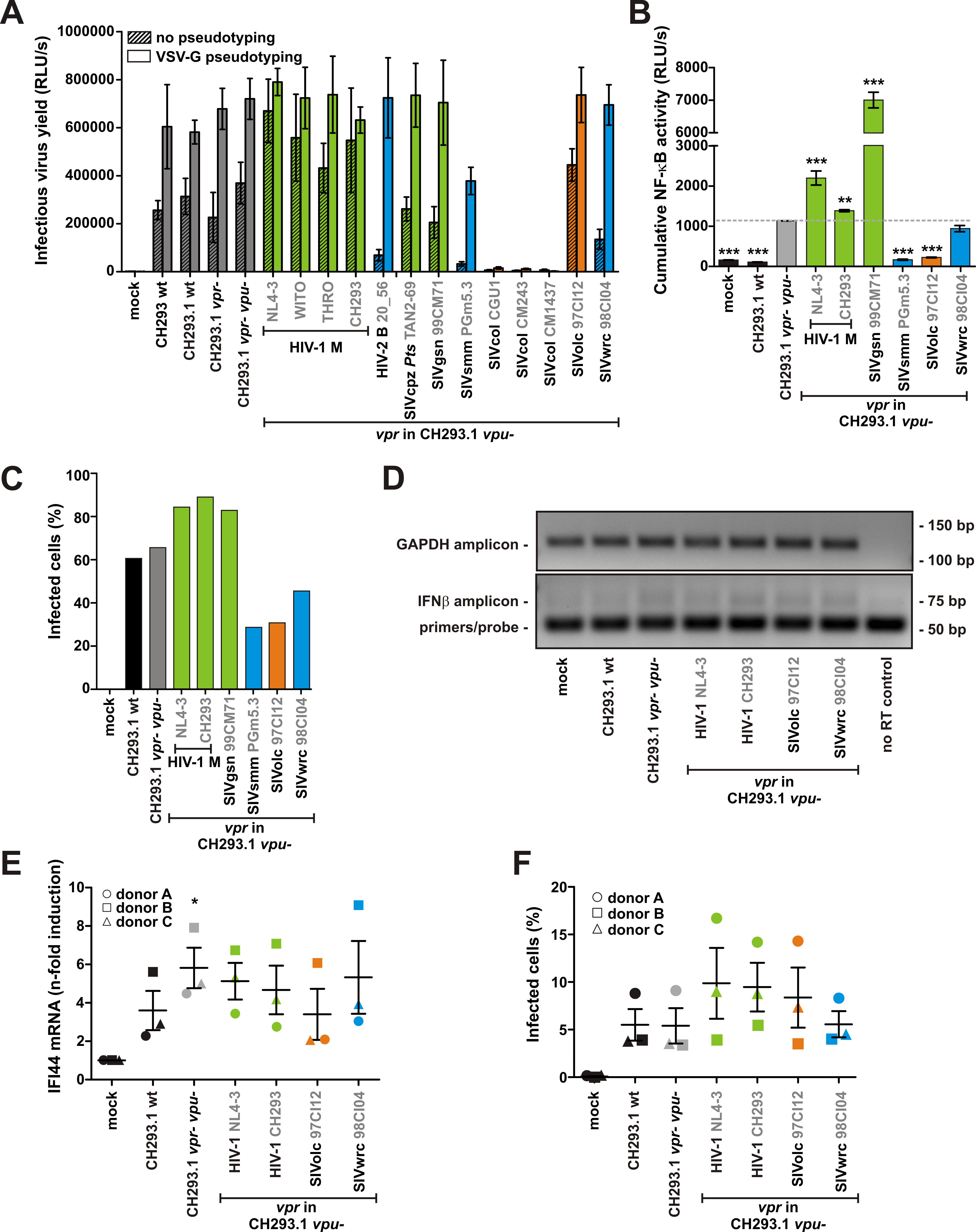

Supplement: S4 Fig — (A) TZM-bl reporter cells were infected with chimeric CH293.1 viruses expressing the indicated vpr alleles. Virus stocks were produced in HEK293T cells and pseudotyped with the glycoprotein of the vesicular stomatitis virus (VSV-G) if indicated. Three days post infection, β-galactosidase activity was determined. Mean values of three experiments with triplicate infections ± SEM are shown. (B) Mean cumulative NF-κB activity of the kinetics shown in Fig 4D was calculated. The mean values of triplicate infections ± SD are shown. Asterisks indicate significant differences compared to CH293.1 vpu- vpr- (**p < 0.01; ***p < 0.001). (C) SupD1 cells were transduced with the indicated VSV-G pseudotyped CH293.1 chimeras. 30 hr post-transduction, the percentage of p24-expressing cells was determined by flow cytometry. Values represent infection rates of the experiment shown in Fig 4D. (D) The GAPDH and IFNβ amplification products of the qRT-PCR analyses shown in Fig 4E of donor B were analyzed by gel electrophoresis. (E) PBMCs were transduced with VSV-G pseudotyped CH293.1 chimeras expressing the indicated vpr alleles. Cells were harvested 72 hr post-transduction, and total cellular RNA was isolated and reversely transcribed. IFI44 mRNA levels were determined by quantitative RT-PCR and normalized to GAPDH mRNA. The mean values ± SEM are shown. Asterisks indicate statistically significant differences compared to CH293.1 wild type infected cells (*p<0.05). (F) The percentage of p24-expressing cells of the experiments shown in Fig 4E and S4E Fig was determined by flow cytometry, 72 hr post-transduction. The results of three donors are shown. Donors A-C in Fig 4E, S4E and S4F Fig are identical. (TIF) [file ppat.1006598.s004.tif]

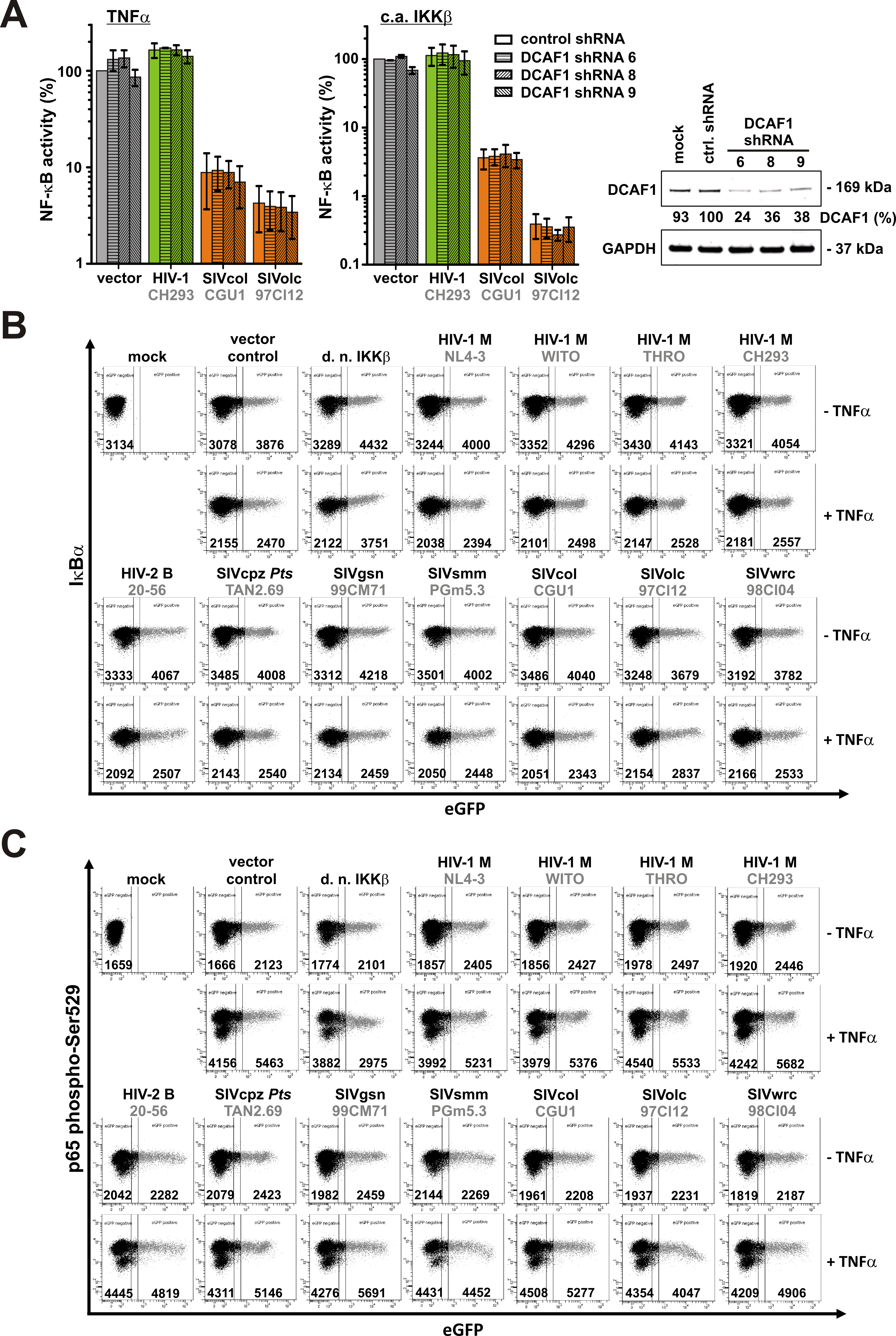

Supplement: S5 Fig — (A) HEK293T cells were cotransfected with the indicated vpr alleles, a firefly luciferase reporter construct under the control of three NF-κB binding sites, a Gaussia luciferase construct for normalization and shRNA vectors to deplete DCAF1. Cells were stimulated with TNFα (left panel) or cotransfected with a constitutively active mutant of IKKβ (c.a. IKKβ) (middle panel). Luciferase activities were determined 40 hr post-transfection. Mean values of two independent experiments performed in triplicate transfections ± SEM are shown. To confirm DCAF1 knockdown, Western blotting of transfected (i.e. eGFP positive cells) was performed 40 hr post-transfection (right panel). GAPDH served as loading control and was used to calculate relative DCAF1 levels. (B) HEK293T cells were transfected with plasmids coexpressing the indicated vpr alleles and eGFP or a dominant-negative mutant of IKKβ (d. n. IKKβ). Cells were stimulated 24 hr post-transfection with TNFα (10 ng/ml) or left untreated. Fifteen minutes after stimulation, cells were harvested, fixed, and permeabilized and levels of IκBα were analyzed by flow cytometry. Primary FACS data of one representative experiment are shown. Numbers indicate the mean fluorescence intensities of IκBα-APC in the eGFP negative and positive populations. (C) Levels of phosphorylated p65 (Ser529) were determined by flow cytometry as described in (B). Primary FACS data of one representative experiment are shown. Numbers indicate the mean fluorescence intensities of p65 phospho-Ser529-APC in the eGFP negative and positive populations. (TIF) [file ppat.1006598.s005.tif]

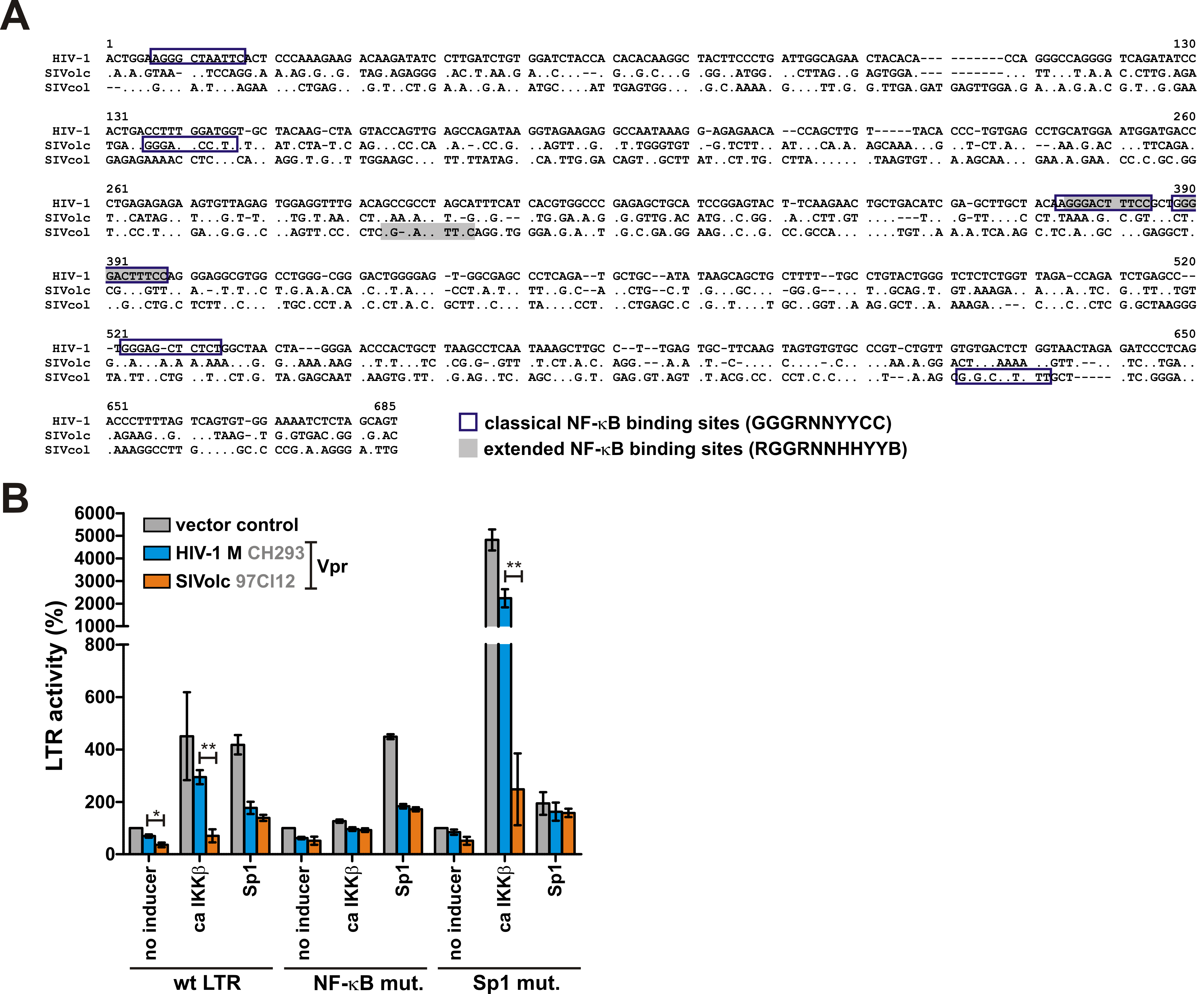

Supplement: S6 Fig — (A) Nucleotide sequence alignment of the HIV-1 NL4-3, SIVcol CM243 and SIVolc 97CI12 LTR sequences analyzed in Fig 6A and 6B. Classical NF-κB binding sites (GGGRNNYYCC) are indicated by blue boxes [60], extended NF-κB binding sites (RGGRNNHHYYB) including sequences bound by RELA homodimers are highlighted in grey [61]. (B) HEK293T cells were cotransfected with the indicated vpr alleles, a firefly luciferase reporter construct under the control of the HIV-1 M LTR promoter, and a Gaussia luciferase construct for normalization. The LTR promoter was either intact (wt) or lacked functional NF-κB or Sp1 binding sites (NF-κB mut. and Sp1 mut., respectively). Cells were stimulated by cotransfection of a constitutively active mutant of IKKβ (c.a. IKKβ) or Sp1. Luciferase activities were determined 40 hr post-transfection. Mean values of three independent experiments in triplicates ± SEM are shown (*p<0.05; **p < 0.01). (TIF) [file ppat.1006598.s006.tif]

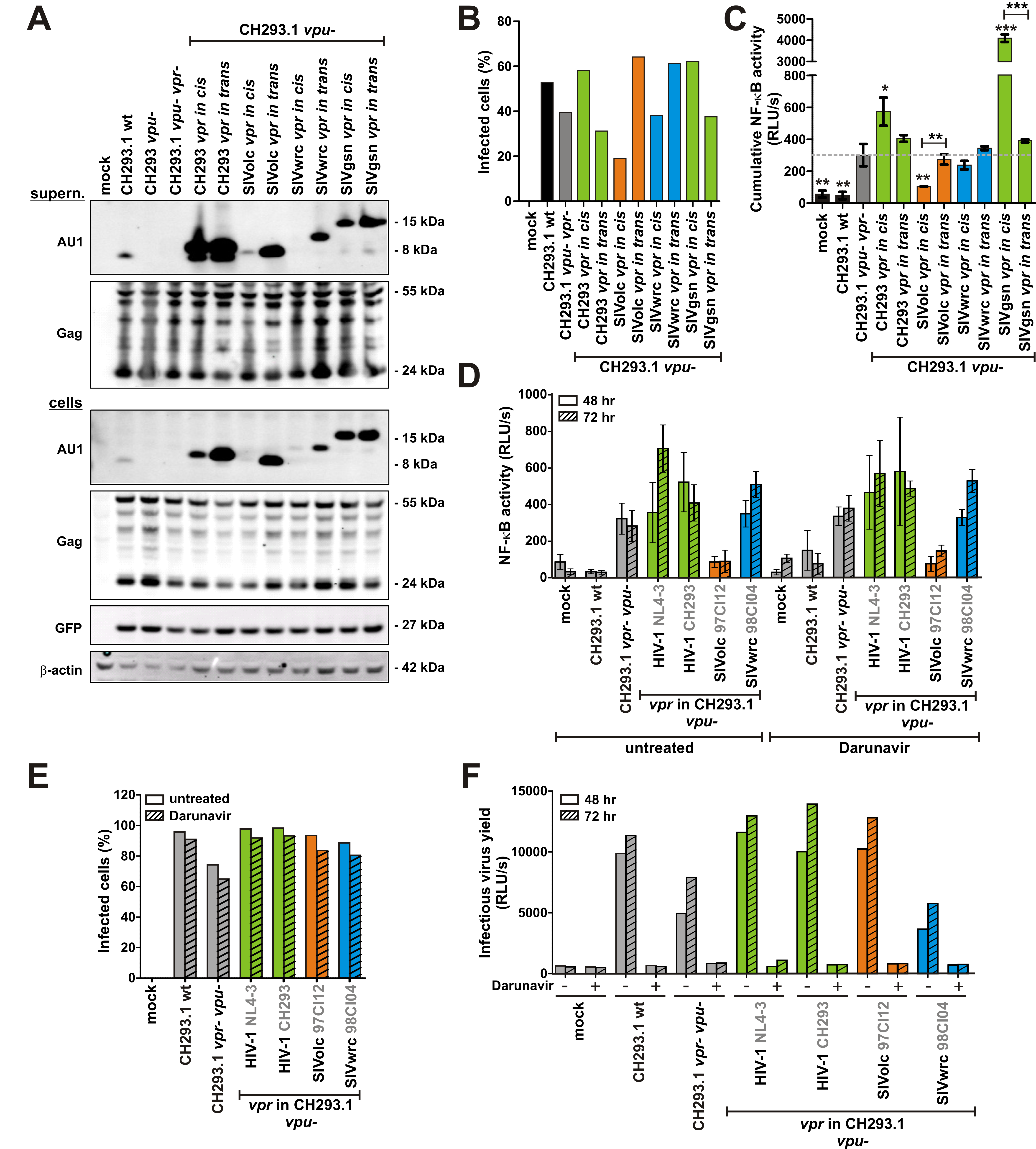

Supplement: S7 Fig — (A) HEK293T cells were transfected with the indicated infectious molecular clones. The indicated vpr alleles were either encoded in the viral genome (in cis) or expressed from a pCG expression vector (in trans) coexpressing eGFP. If Vpr was expressed in cis, a vector expressing only eGFP was cotransfected. 40 hr post-transfection, cells and supernatants were harvested and Western blotting was performed to detect AU1-tagged Vpr and Gag. eGFP and β-actin were detected to control for transfection efficiencies and protein loading, respectively and served as purity controls in the supernatant. (B) SupD1 cells were transduced with the indicated VSV-G pseudotyped CH293.1 viruses expressing Vpr in cis or in trans. 30 hr post-transduction, the percentage of p24-expressing cells was determined by flow cytometry. Values represent infection rates of the experiment shown in Fig 7. (C) Mean cumulative NF-κB activity of the kinetics shown in Fig 7 was calculated. The mean values of triplicate infections ± SD are shown. Asterisks indicate significant differences compared to CH293.1 vpu- vpr- (*p<0.05; **p < 0.01; ***p < 0.001). (D) SupD1 cells were transduced with the indicated VSV-G pseudotyped CH293.1 mutants in the presence or absence of the protease inhibitor Darunavir (100 nM). As a reporter for NF-κB activation, firefly luciferase activities in the cells were determined 48 and 72 hr after transduction. The mean values of triplicate infections ± SD are shown. (E) Infection rates of the cells described in (D) were determined 48 hr after infection by p24 staining in permeabilized cells, followed by FACS analysis. (F) Supernatants of the SupD1 cells described in (D) were harvested 48 and 72 hr after infection and infectious virus yield was determined by infection of TZM-bl cells for 72 h in the presence of Darunavir (100 nM). In (D), (E) and (F), the results of the same representative experiment are shown. (TIF) [file ppat.1006598.s007.tif]
