# Supplementary material for: Primate lentiviruses use at least three alternative strategies to suppress NF-κB-mediated immune activation
Source: PLoS Pathog. 2017 Aug 31;13(8):e1006598. doi: 10.1371/journal.ppat.1006598 (PMC5597281; doi:10.1371/journal.ppat.1006598)
Supplement: S1 Table — (DOCX) [file ppat.1006598.s008.docx]

**S1 Table. Oligonucleotides used to generate HIV-1 NL4-3 IRES eGFP constructs expressing different *nef* alleles.**

| **number** | **designation** | **oligonucleotide sequence (5`- 3`)** |
| --- | --- | --- |
| P1 | SIVcol *nef* seq. fw | agtgggaacagccagacaacg |
| P2 | SIVcol *nef* seq. rev | gtccctcccaacttggaaagtccc |
| P3 | HpaI env CM243 nef fw | gctgttaacttgctcaatgccacagcc |
| P4 | HpaI env CM243 nef rev | cttatagcaaaatcctttccaagccc |
| P5 | CM243 nef MluI fw | gggcttggaaaggattttgctataagatgggctccatactgtcc |
| P6 | CM243 nef MluI rev | ctacgcgtcagtccaccatctctatggc |
| P7 | HpaI env CM1437 nef fw | gctgttaacttgctcaatgccacagcc |
| P8 | HpaI env CM1437 nef rev | cttatagcaaaatcctttccaagccc |
| P9 | CM1437 nef MluI fw | gggcttggaaaggattttgctataagatgggatccatactgtcatgc |
| P10 | CM1437 nef MluI rev | ctacgcgtcagtccaccatctctatggcaac |
