# Supplementary material for: Primate lentiviruses use at least three alternative strategies to suppress NF-κB-mediated immune activation
Source: PLoS Pathog. 2017 Aug 31;13(8):e1006598. doi: 10.1371/journal.ppat.1006598 (PMC5597281; doi:10.1371/journal.ppat.1006598)
Supplement: S2 Table — (DOCX) [file ppat.1006598.s009.docx]

**S2 Table. Oligonucleotides used to generate pCG IRES eGFP constructs expressing different *vpr* alleles.**

| **number** | **designation** | **oligonucleotide sequence (5`- 3`)** |
| --- | --- | --- |
| P11 | SIVcol *vpr* seq. fw | ggcttaccctggaatccttgtg |
| P12 | SIVcol *vpr* seq. rev | ccttttgaaagaaagcatagctgacagtg |
| P13 | CM243/CM1437 N-AU1 *vpr* fw | cgtctagaatatggacacctacaggtacatctcttctggccctaagagagaaag |
| P14 | CM243/CM1437 *vpr* rev | ctacgcgtctaatggtcttgtctagggggaaag |
