# Supplementary material for: Primate lentiviruses use at least three alternative strategies to suppress NF-κB-mediated immune activation
Source: PLoS Pathog. 2017 Aug 31;13(8):e1006598. doi: 10.1371/journal.ppat.1006598 (PMC5597281; doi:10.1371/journal.ppat.1006598)
Supplement: S3 Table — (DOCX) [file ppat.1006598.s010.docx]

**S3 Table. Oligonucleotides used to generate CD8-CD3ζ fusion constructs.**

| **number** | **designation** | **oligonucleotide sequence (5`- 3`)** |
| --- | --- | --- |
| P15 | CD3ζ UTR 1 fw | cagcctctgcctcccaacctc |
| P16 | CD3ζ UTR 1 rev | gcaggtctggcctttgagtgg |
| P17 | CD3ζ UTR 2 fw | ccaacctctttctgagggaaagg |
| P18 | CD3ζ UTR 1 rev | ctttgagtggtgaaatcccctgg |
| P19 | CD3ζ seq1F | caatctaggacgaagagaggag |
| P20 | CD3ζ seq1R | ccgccatcttatctttctgcag |
