# Supplementary material for: Primate lentiviruses use at least three alternative strategies to suppress NF-κB-mediated immune activation
Source: PLoS Pathog. 2017 Aug 31;13(8):e1006598. doi: 10.1371/journal.ppat.1006598 (PMC5597281; doi:10.1371/journal.ppat.1006598)
Supplement: S4 Table — (DOCX) [file ppat.1006598.s011.docx]

**S4 Table. Oligonucleotides used to generate pGL3 LTR firefly luciferase reporter constructs.**

| **number** | **designation** | **oligonucleotide sequence (5`- 3`)** |
| --- | --- | --- |
| P21 | SIVcol CM243 3’LTR seq. fw | tggaggggattattagaacccctgag |
| P22 | SIVcol CM243 3’LTR seq. fw | gatcagtctcctcttcttcttccgc |
| P23 | SIVcol CM243 3’LTR MluI fw | gaacgcgttggaggggattattagaacccctgag |
| P24 | SIVcol CM243 3’LTR XhoI rev | cttactcgagcaagtccctgttcgggcg |
