# Supplementary material for: Primate lentiviruses use at least three alternative strategies to suppress NF-κB-mediated immune activation
Source: PLoS Pathog. 2017 Aug 31;13(8):e1006598. doi: 10.1371/journal.ppat.1006598 (PMC5597281; doi:10.1371/journal.ppat.1006598)
Supplement: S5 Table — (DOCX) [file ppat.1006598.s012.docx]

**S5 Table. Oligonucleotides used to generate CH293.1 proviruses expressing heterologous *vpr* alleles.**

| **number** | **designation** | **oligonucleotide sequence (5`- 3`)** |
| --- | --- | --- |
| P25 | HpaI fw | gtaaaacagttaacaggggcag |
| P26 | AfeI rev | gtcttctgctctttccctaattc |
| P27 | del *vpr* XbaI/MluI fw | gtctagagcaacgcgtcatggagccagtagatcctaacctag |
| P28 | del *vpr* XbaI/MluI rev | gacgcgttgctctagactagtgtccattcattgtatggttccctctgcggcccctggtcctctgggacttgttccacctatcctc |
| P29 | PsiI fw | tttctattgcaatacatcaaaac |
| P30 | XmaI rev | ccggctcgtatgttg |
| P31 | XbaI silencing fw | ctgtttgatagcttagcaataacagtagctgaagggacagatagaattcttgaag |
| P32 | XbaI silencing rev | gttattgctaagctatcaaacagactaatagcacttttctttagctcaagacc |
| P33 | *vpu* stop fw | atcttatatcaaagcagtaagtactaagtagtatatgtaatgtaatgatggatagaaaaagcagattata |
| P34 | *vpu* stop rev | tataatctgctttttctatccatcattacattacatatactacttagtacttactgctttgatataagat |
